# Prevalence of syphilis among people living with HIV and its implication for enhanced coinfection monitoring and management in China: A meta-analysis

**DOI:** 10.3389/fpubh.2022.1002342

**Published:** 2022-10-17

**Authors:** Yuelin Wu, Wenqian Zhu, Chengqing Sun, Xiaoli Yue, Min Zheng, Gengfeng Fu, Xiangdong Gong

**Affiliations:** ^1^Institute of Dermatology, Chinese Academy of Medical Sciences and Peking Union Medical College, Nanjing, China; ^2^School of Public Health, Nanjing Medical University, Nanjing, China; ^3^Department of STD Epidemiology, National Center for STD Control, Nanjing, China; ^4^Department of HIV/STD Control and Prevention, Guizhou Provincial Center for Disease Control and Prevention, Guiyang, China; ^5^Department of HIV/STD Control and Prevention, Jiangsu Provincial Center for Disease Control and Prevention, Nanjing, China

**Keywords:** HIV, syphilis, prevalence, coinfection, meta-analysis

## Abstract

**Background:**

People living with HIV (PLWH) are at an increased risk of syphilis infection. The objectives of this study were to assess the overall prevalence of syphilis among PLWH in China and identify factors associated with syphilis infection among PLWH.

**Methods:**

We searched Medline, Embase, China National Knowledge Infrastructure (CNKI), Chinese Scientific Journals Database (VIP), Wan-fang Data, and Chinese Biomedical Literature Database (CBM) to identify studies that reported the prevalence of syphilis among PLWH in China and were published in English or Chinese from January 1, 1990, to May 31, 2022. The reference lists of retrieved articles and relevant reviews were also checked to identify additional studies. A random-effect model was fitted to calculate the pooled syphilis prevalence among PLWH. Subgroup analyses, meta-regression analyses and sensitivity analyses were conducted to determine the potential source of heterogeneity.

**Results:**

Of the 1,599 articles screened, 29 studies involving 34,740 participants were eligible for inclusion in this meta-analysis. The overall prevalence of syphilis among PLWH in China was 19.9% [95% confidence interval (CI): 15.4–24.8%, *I*^2^ = 98.9%]. Subgroup analysis showed that the pooled prevalence of syphilis among men who have sex with men (MSM) with HIV (21.9%, 95% CI: 17.2–26.9%) was much higher than that among heterosexuals (10.3%, 95% CI: 5.2–16.8%); there was regional diversity in the prevalence of syphilis, the highest in northern China (31.7%, 95% CI: 17.9–47.4%), followed by central-southern China (26.7%, 95% CI: 11.4–45.7%), and the lowest in northwestern China (15.0%, 95% CI: 6.9–25.4%); the syphilis prevalence among PLWH decreased as CD4 + T cell count increased (19.6% in CD4 + T cell < 200 vs. 8.7% in ≥ 500) and was higher among non-antiretroviral therapy (non-ART) HIV-infected patients (21.0%, 95% CI: 9.9–35.0%) than that among ART ones (16.1%, 95% CI: 3.9–34.3%).

**Conclusions:**

Our study showed a significantly high prevalence of syphilis among PLWH in China, particularly among MSM with HIV. Developing national guidelines for the integrated screening, monitoring, and management of HIV and syphilis as well as syphilis diagnosis and treatment training programs for physicians at designated HIV treatment hospitals is urgent and crucial to combat HIV and syphilis coinfection in China.

## Introduction

Syphilis is a sexually transmitted disease (STD) caused by *Treponema pallidum*, a subspecies pallidum. It affects various systems of the human body, leading to clinical chronic manifestations such as neurosyphilis, ocular syphilis, otosyphilis, and cardiovascular syphilis (1, 2). Syphilis is known as a serious public health threat worldwide, with an estimate of 7.1 million new cases in 2020 (3). In China, the number of reported syphilis cases has been increasing since the resurgence of syphilis in the late 1970s and remained the third among class B national notifiable infectious diseases since 2009 (4, 5), which has become a major disease burden. In recent years, the incidence of syphilis kept rising, the case rate increased from 31.85 per 100,000 to 38.37 per 100,000 during 2015 and 2019 (6).

The epidemic of HIV is also a serious public health concern in China. HIV has spread from drug users to men who have sex with men (MSM), and to the general urban population, the transmission pattern has changed from intravenous injection through shared syringes to sexual transmission, as a real sexually transmitted infection (7). The high-risk and vulnerable groups of HIV are the same as syphilis, such as MSM, female sex workers (FSWs), college students, and the elderly (8). The national HIV/AIDS sentinel surveillance showed that the HIV prevalence among MSM in China has been on the rise, from 3.0 to 8.0% during 2006 and 2015 (9), and the number of newly diagnosed college students has seen an annual growth rate ranging from 30 to 50% over the past several years (7). The number of reported HIV cases in China increased from 50,330 in 2015 to 71,204 in 2019 (10), with an increase of 41.5%. The Chinese Center for Disease Control and Prevention (Chinese CDC) estimated that by the end of 2020, there were 1.05 million people living with HIV (PLWH), and 351,000 AIDS-related deaths, representing a severe disease burden in the country (8).

The bidirectional interaction between syphilis and HIV has been referred to as “epidemiological synergy” (11). Studies confirmed that syphilis could facilitate the transmission and acquisition of HIV infection, increasing infectiousness through effects on HIV shedding, HIV replication, and increases in viral diversity, and increasing susceptibility by mucosal disruption, immune changes in the genital tract and effects on the genital tract microenvironment (12–14). In turn, HIV could alter the syphilis manifestations and blur the distinction of the stages (12). HIV and syphilis coinfection patients also face a higher risk of treatment failure and the emergence of neurosyphilis (15).

Due to the reciprocal synergistic interaction between syphilis and HIV, the rising epidemic of this concomitant syphilis and HIV infection remains hard to manage. At present, China faces the double challenge of syphilis and HIV epidemic. In order to better control the prevalence of syphilis and HIV, it is an important option to take their coinfection as the entry point for prevention and treatment, and there is of great significance to strengthen the monitoring and treatment of syphilis in HIV infections. However, the coinfection of syphilis and HIV has not received enough attention in China, indicating a gap in the prevention and control of syphilis and HIV. To fill the gap and to address HIV and syphilis coinfection, primarily we need to determine the prevalence of syphilis among PLWH. The objectives of this meta-analysis were to determine the overall prevalence of syphilis among PLWH in China and identify factors associated with syphilis among PLWH.

## Methods

### Search strategy

We searched eligible studies that reported syphilis prevalence among PLWH published in English or Chinese within each of the following databases: Medline (Ovid & PubMed interface), Embase, China National Knowledge Infrastructure (CNKI), Chinese Scientific Journals Database (VIP), Wan-fang Data, and Chinese Biomedical Literature Database (CBM) from January 1, 1990, to May 31, 2022. The search was conducted by using free-text terms and Medical Subject Headings (MeSH) terms that combined “human immunodeficiency virus,” “syphilis,” “co-infection,” and “China” in the international databases, and equivalent terms in Chinese in domestic databases. The search strategies are given in details in Supplementary Table 1. Reference lists of the included articles and relevant reviews were also checked to identify additional publications. The Preferred Reporting Items for Systematic Reviews and Meta-Analyses (PRISMA) statement was used to report the results of this meta-analysis (16).

### Inclusion and exclusion criteria

Publications were included if they met the following criteria: (1) study was conducted in China, (2) the prevalence of syphilis among PLWH was reported, (3) study participants aged 15 years or older or age-specific syphilis prevalence was available, (4) HIV and syphilis infections were confirmed by standard laboratory serologic testing, and (5) the number of HIV-infected participants was more than 300, for getting the stable and reliable results based on sample size calculation (17). If multiple publications were reported from the same study, only one with the most comprehensive reporting or the largest sample size was included.

### Study selection and quality assessment

For studies returned by the search strategy, we created an Endnote library (version 20.1) to catalog the search results and de-duplicate references. After removing duplicates, two reviewers (YW and WZ) independently screened the title and abstract of all the records. If a study could not be definitively excluded based on its title and abstract, its full text was obtained for thorough screening. We then screened the full text for inclusion and assessed the quality of the included studies. For excluded studies, we recorded the explicit reason of exclusion for each study (Supplementary Table 2). We also included dissertations and conference abstracts if eligible. Disagreements were resolved through discussion between the two reviewers, and, if necessary, consultation with the third reviewer (XG). Study quality was assessed by Loney's 8-item scale (each item was assigned a score of 1 point, totaling 8 points): study design and sampling method, sampling frame, sample size, appropriate measurement, unbiased measurement, response rate, estimates of prevalence, and description of study subjects (17) (Supplementary Table 3). A score of 7–8 is considered high quality, 4–6 moderate quality, and 0–3 low quality.

### Data extraction

A standardized data extraction form was developed and piloted specifically for this study. Information was extracted by the two reviewers (YW and WZ) in five aspects: (1) basic information (e.g., first author, publication year, publication language, study location, study period, recruitment site, sampling method, and sample size), (2) the prevalence of syphilis among PLWH and the most likely transmission route of HIV, (3) HIV and syphilis testing methods, (4) sociodemographic characteristics of PLWH, e.g., age and sex, and (5) history of diagnosis and treatment of HIV.

### Statistical analysis

After Freeman-Tukey double arcsine transformation to stabilize the variances (18), a random-effects model meta-analysis was performed to estimate the pooled prevalence of syphilis and its 95% confidence interval (CI), because there was substantial heterogeneity across studies. The *I*^2^ statistic (values of 25, 50, and 75% are considered to represent low, medium, and high heterogeneity, respectively) was calculated to assess the heterogeneity of the studies (19).

Subgroup analyses were conducted to assess the associations of sample size, publication language, study quality, recruitment site, study region, study period, transmission category, sex, age, CD4 + T cell count, and antiretroviral therapy (ART) with the prevalence of syphilis among PLWH. Differences between subgroups were analyzed using the Q test based on the fixed-effects model (20). We also performed random-effects multivariable meta-regression analyses to examine the possible sources of heterogeneity with the following covariates: sample size, publication language, study quality, recruitment site, and study region.

Potential publication bias was assessed by visual inspection of the funnel plot and statistical Egger's test (a *p*-value < 0.10 was considered statistically significant) (21). Sensitivity analyses were conducted with the leave-one-out method or finding outliers of syphilis prevalence and omitting the corresponding study.

All statistical analyses were performed using R studio (version 1.4.1717) with the package meta (version 4.19-0).

## Results

### Study selection

**Figure 1:**
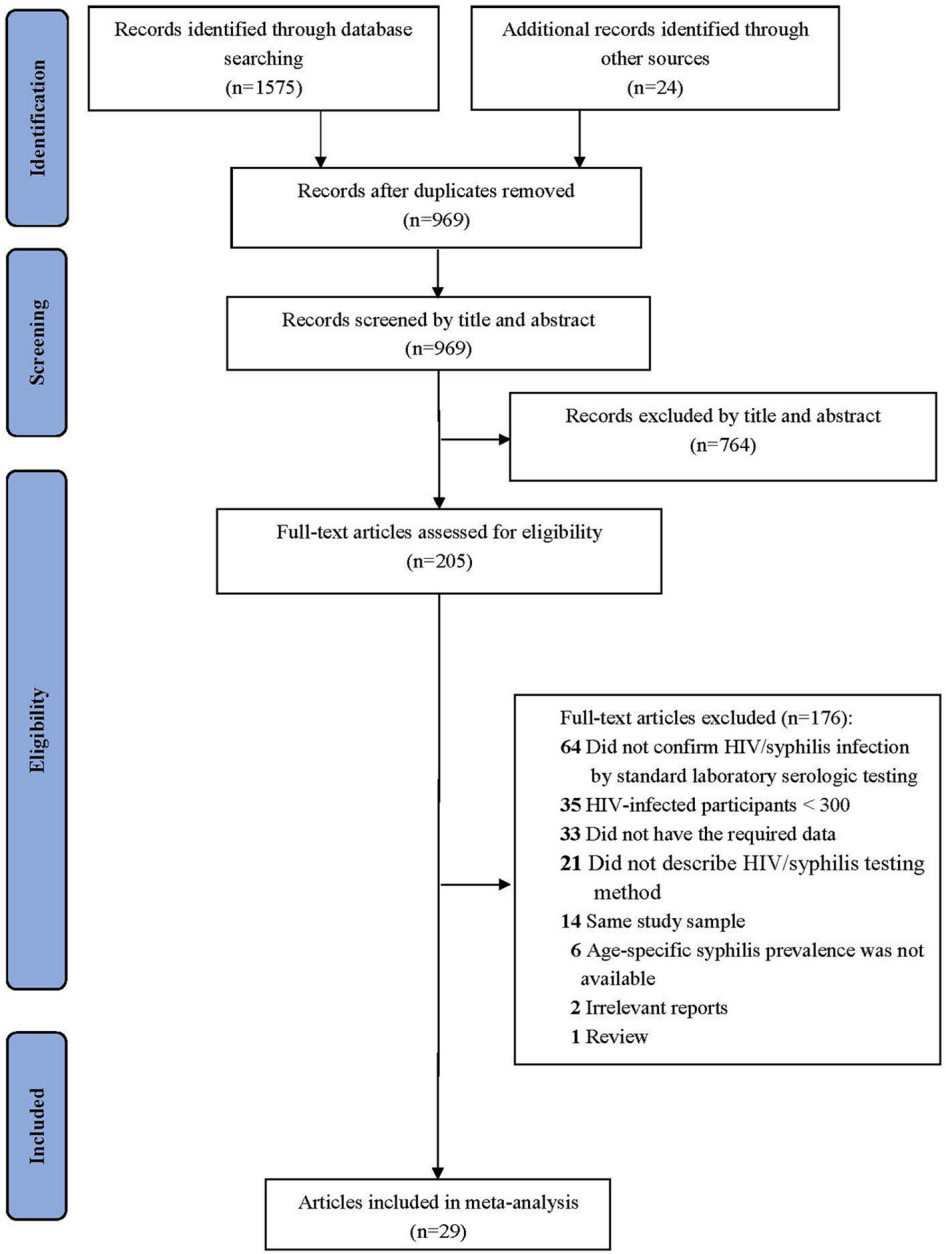
Flow diagram of study selection.

A total of 1,599 studies were identified by the initial search. After removing 630 duplicates, we screened the remaining 969 studies by reviewing their title and abstract. A total of 205 full-text articles were assessed for eligibility, of which 176 were excluded (description of excluded studies is recorded in Supplementary Table 2). Finally, 29 studies were included in this meta-analysis (22–50). The flow chart of our systematic literature search is presented in Figure 1.

### Study characteristics

The characteristics of the included studies are presented in Table 1. Of these studies, eight studies (28%) were published in English and 21 studies (72%) in Chinese, covering 67 cities from 30 provinces and municipalities in mainland China (no studies were identified in Tibet). There were no studies of low quality (Table 1; Supplementary Table 4). The total number of PLWH study participants was 34,740.

**Table 1:** Characteristics of included studies.

**References**	**Study period**	**Study location**	**Recruitment site**	**Study design**	**Sampling method**	**HIV testing method**	**Syphilis testing method**	**Sample size**	**Quality assessment score**
Zhang et al. (22)	2006–2009	Chongqing	Community	Cross-sectional study	Snowball sampling	ELISA + WB	RPR + TPPA	472	6
Gao et al. (23)	2011	Guangdong	Local CDC	Cross-sectional study	Successive sampling	WB	ELISA + TRUST	1,573	8
Wang et al. (24)	2009–2012	Shanghai	Infectious disease hospital	Cross-sectional study	Successive sampling	WB	TRUST + TPPA	686	4
Wu et al. (25)	2004–2011	Zhejiang	Local CDC	Cross-sectional study	Successive sampling	WB	ELISA/TPPA + RPR	460	8
Wu et al. (26)	2014–2016	Heilongjiang, Jilin, Liaoning, et al.	Community	Cross-sectional study	Snowball sampling + RDS	ELISA + WB	RPR/TRUST + TPPA	2,314	6
Hu et al. (27)	2009–2013	Liaoning	Infectious disease hospital	Retrospective cohort study	Successive sampling	ELISA + WB	RPR + TPPA	1,010	7
Li et al. (28)	2011–2013	Beijing	Local CDC	Cross-sectional study	Successive sampling	WB	RPR + TPPA	3,690	8
Li et al. (29)	2013–2014	Beijing	Infectious disease hospital	Cross-sectional study	Successive sampling	WB	TPPA + RPR	1070	6
Ya et al. (30)	2010–2015	Jiangsu	VCT clinic	Cross-sectional study	Successive sampling	HIV antibody testing + WB	RPR + TPPA	421	8
Ma et al. (31)	2011–2014	Liaoning	Local CDC	Cross-sectional study	Successive sampling	WB	TRUST + TPPA	1,653	8
Cao et al. (32)	2002–2014	Ningxia	Local CDC	Cross-sectional study	Successive sampling	WB	ELISA + TRUST	703	7
Yuan et al. (33)	2015	Zhejiang	Local CDC	Cross-sectional study	Successive sampling	WB	RPR + TPHA	2,090	7
Wang et al. (34)	2007–2016	Tianjin	STD clinic	Cross-sectional study	Successive sampling	ELISA + WB	TPPA + RPR	452	6
Hu et al. (35)	2009–2014	Liaoning	Infectious disease hospital	Cross-sectional study	Successive sampling	ELISA + WB	RPR + TPPA	545	7
Liu et al. (36)	2016	Shanghai and Shandong	Infectious disease hospital	Cross-sectional study	Convenience sampling	WB	ELISA + TRUST	380	5
Fan et al. (37)	2014	Sichuan	Community	Cross-sectional study	Convenience sampling	WB	TPPA + TRUST	433	5
Zhou et al. (38)	2010–2016	Chongqing	Community	Cross-sectional study	Snowball sampling + RDS	ELISA + WB	ELISA + RPR	1,537	6
Liu et al. (39)	2017	Beijing	Infectious disease hospital	Cross-sectional study	Successive sampling	WB	RPR + TPPA	385	7
Zhao et al. (40)	2011–2016	Xinjiang	Local CDC	Cross-sectional study	Successive sampling	HIV antibody testing + WB	RPR + TPPA	567	8
Wu et al. (41)	2013–2017	Jiangsu	Local CDC	Cross-sectional study	Successive sampling	WB	RT + RPR	3,444	8
Weng et al. (42)	2009–2017	Guangdong	STD clinic	Cross-sectional study	Convenience sampling	ELISA + WB	TRUST + TPPA	561	7
Gao et al. (43)	2010–2017	Jiangsu	VCT clinic	Cross-sectional study	Successive sampling	ELISA + WB	RPR + TPPA	639	5
Zhu et al. (44)	2008–2009	Zhejiang	Local CDC	Cross-sectional study	Successive sampling	WB	ELISA + TRUST	1,199	5
Wang et al. (45)	2013–2016	Shanxi	Community	Cross-sectional study	Snowball sampling	ELISA + WB	TRUST + TPPA	325	5
Li et al. (46)	2009–2015	Yunnan	Local CDC	Retrospective cohort study	Successive sampling	ELISA + WB	TRUST + TPPA	2,162	5
Sun et al. (47)	2015–2018	Guangdong	Infectious disease hospital	Cross-sectional study	Successive sampling	WB	TPPA + RPR	4,493	8
Zhang et al. (48)	2009–2018	Heilongjiang	Community	Cross-sectional study	Snowball sampling	ELISA + WB	ELISA + RPR	543	4
Cheng et al. (49)	2019–2020	Beijing	Infectious disease hospital	Cross-sectional study	Successive sampling	ELISA + WB	TPPA + RPR	527	5
Tu et al. (50)	2020–2021	Yunnan	Infectious disease hospital	Cross-sectional study	Cluster sampling	WB	TPPA + TRUST	406	5

CDC, Center for Disease Control and Prevention; ELISA, Enzyme linked immunosorbent assay; RDS, Respondent-driven sampling; RPR, Rapid plasma regain; RT, Rapid immunochromatography test; STD, Sexually transmitted disease; TPHA, Treponema pallidum hemagglutination; TPPA, Treponema pallidum particle agglutination; TRUST, Toluidine red unheated serum test; VCT, Voluntary counseling and testing; WB, Western blot.

### Pooled prevalence of syphilis among PLWH

**Figure 2:**
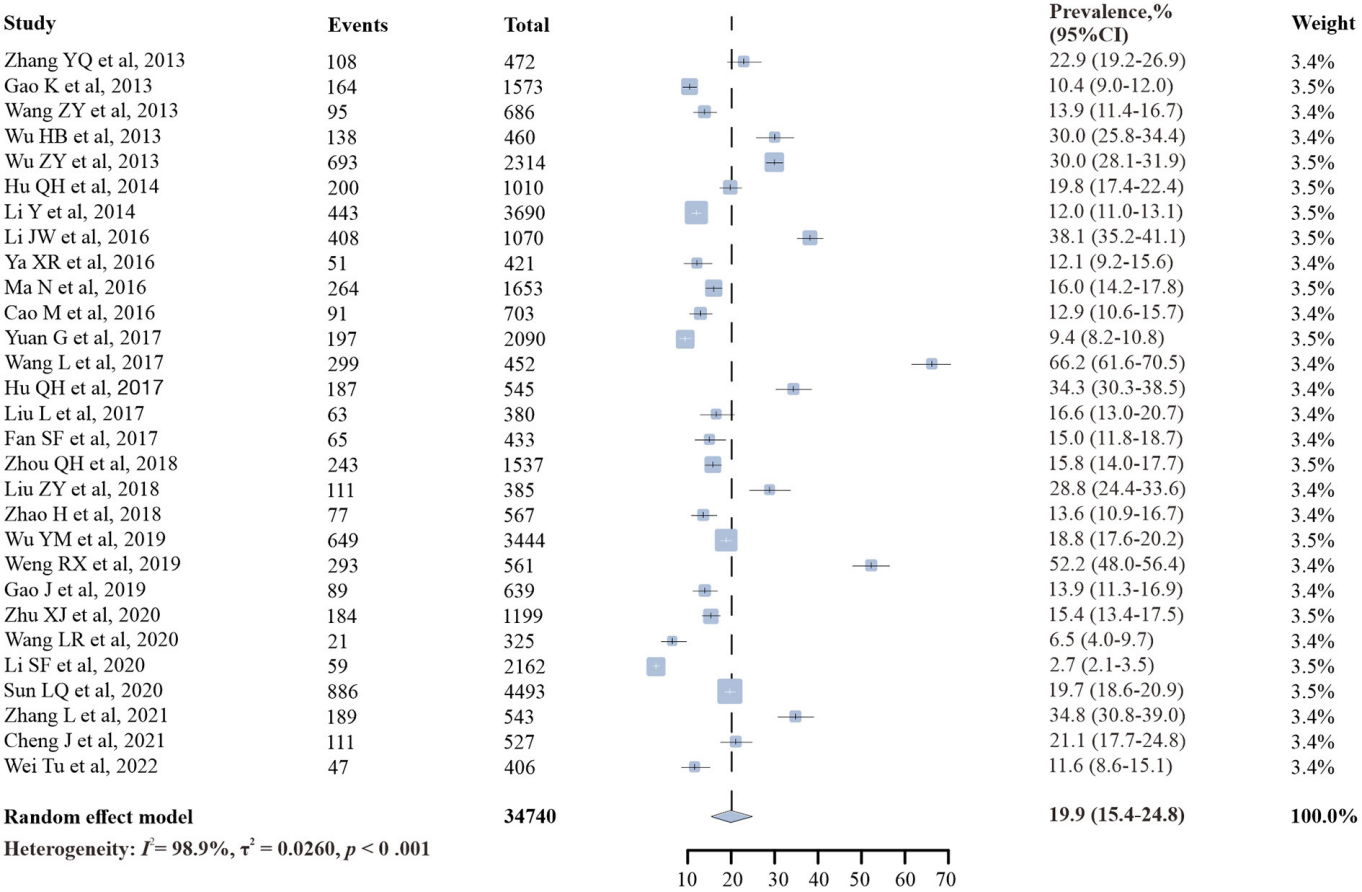
Forest plots of pooled prevalence of syphilis among people living with HIV.

The prevalence of syphilis among PLWH in China ranged from 2.7 to 66.2%, and the overall random-effects pooled prevalence was 19.9% (95% CI: 15.4–24.8%), with substantial heterogeneity (*I*^2^ = 98.9%, *p* < 0.001) (Figure 2).

### Subgroup analysis

**Figure 3:**
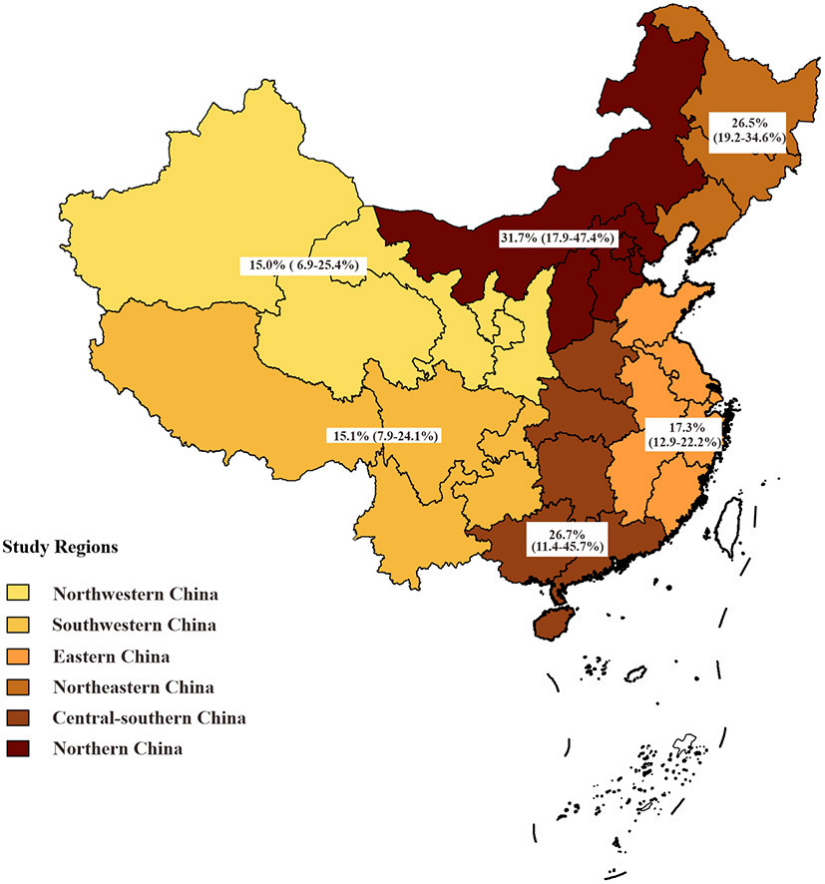
Prevalence of syphilis among people living with HIV by study region.

Subgroup analysis showed that syphilis prevalence among PLWH attending STD clinics was significantly higher (59.2%, 95% CI: 45.4–72.4%) than that of other PLWH. The pooled prevalence of syphilis among PLWH was the highest in northern China (31.7%, 95% CI: 17.9–47.4%), followed by central-southern China (26.7%, 95% CI: 11.4–45.7%), and the lowest in northwestern China (15.0%, 95% CI: 6.9–25.4%) (Table 2; Figure 3). Despite some fluctuations over time, the prevalence of syphilis among PLWH in China remained high (Table 2).

**Table 2:** Subgroup analysis of factors associated with syphilis prevalence among people living with HIV.

**Subgroup analysis**	**Studies, No**.	**Prevalence, % (95% CI)**	**I^2^, %**	***p*** **-values**	
				**Subgroup difference**	**Egger test**
**Sample size**					
< 600	14	24.8 (16.7–33.8)	98.5	< 0.001	0.13
≥600	15	15.8 (11.8–20.2)	98.9		>0.99
**Publication language**					
Chinese	21	19.7 (15.0–25.0)	98.4	0.13	0.17
English	8	20.4 (10.1–33.1)	99.4		0.58
**Study quality**					
Moderate	15	20.1 (13.1–28.1)	99.2	< 0.001	0.49
High	14	19.8 (14.3–25.9)	98.4		0.19
**Recruitment site**					
Local CDC	10	13.4 (9.3–18.1)	98.4	< 0.001	0.62
Infectious disease hospital	9	22.1 (16.5–28.3)	97.1		0.78
VCT clinic	2	13.2 (11.2–15.3)	0.0		Not estimable^a^
STD clinic	2	59.2 (45.4–72.4)	95.0		Not estimable^a^
Community	6	20.0 (12.1–29.2)	97.9		0.40
**Study region** ^b^					
Northeast	5	26.5 (19.2–34.6)	97.7	< 0.001	0.64
North	6	31.7 (17.9–47.4)	99.4		0.15
Northwest	4	15.0 (6.9–25.4)	98.6		0.014
East	9	17.3 (12.9–22.2)	97.9		0.71
Central-south	4	26.7 (11.4–45.7)	99.4		0.52
Southwest	6	15.1 (7.9–24.1)	99.4		0.93
**Study period** ^b^					
2000–2010	14	24.1 (15.8–33.7)	99.3	< 0.001	0.11
2011–2021	27	19.5 (14.7–24.7)	98.9		0.10
**Transmission category**					
Sexual contact	19	20.4 (15.4–25.9)	98.7	< 0.001	0.48
Heterosexual	5	10.3 (5.2–16.8)	98.3		0.45
Male-to-male	19	21.9 (17.2–26.9)	97.3		0.84
IDU	3	9.5 (1.4–22.9)	87.2		0.33
Unknown	4	13.5 (9.8–17.6)	0.0		0.61
**Sex**					
Female	6	6.0 (3.1–9.6)	87.8	< 0.001	0.42
Male	20	20.1 (15.3–25.3)	98.5		0.80
**Age, y**					
< 50	6	24.2 (11.4–40.0)	99.4	< 0.001	0.024
≥50	6	31.8 (11.5–56.4)	97.8		0.036
**CD4 + T cell count, cells/ mm** ^**3**^					
< 200	6	19.6 (8.8–33.3)	98.0	< 0.001	0.006
200–349	6	14.7 (7.4–24.0)	96.7		0.002
350–499	3	9.1 (2.9–18.2)	95.2		0.66
≥500	3	8.7 (2.4–18.4)	93.5		0.60
**ART**					
No	3	21.0 (9.9–35.0)	96.0	< 0.001	0.17
Yes	3	16.1 (3.9–34.3)	94.0		0.24

ART, Antiretroviral therapy; CDC, Center for Disease Control and Prevention; IDU, Intravenous drug use; STD, Sexually transmitted disease; VCT, Voluntary counseling and testing.

a Because of insufficient observations.

b One study could be included in more than one category for this variable; for example, if the study was done from 2000 to 2014, it would be included in both the 2000–2010 and 2011–2021 categories.

Nineteen studies (22, 25–27, 30, 31, 35–39, 42–49) reported the prevalence of syphilis among PLWH whose most likely transmission route was male-to-male sexual contact, five studies among PLWH with heterosexual transmission (27, 44, 46, 47, 49), and three studies (44, 46, 49) among PLWH who reported intravenous drug use (IDU). The prevalence of syphilis was found to be the highest among PLWH who reported male-to-male sexual contact (21.9%, 95% CI: 17.2–26.9%). We included 20 studies (22, 25–27, 30, 31, 33, 35–39, 42–49) that reported the prevalence of syphilis among PLWH by sex. The prevalence of syphilis among males living with HIV (20.1%, 95% CI: 15.3–25.3%) was significantly higher than that among females (6.0%, 95% CI: 3.1–9.6%). The analysis by age showed that the prevalence of syphilis among PLWH was significantly higher in those aged ≥ 50 years (31.8%, 95% CI: 11.5–56.4%) than in those aged < 50 years (24.2%, 95% CI: 11.4–40.0%). When stratified by CD4 + T cell count, we found that syphilis prevalence among PLWH decreased as CD4 + T cell count increased. Subgroup analysis by ART status showed higher syphilis prevalence among non-treated HIV-infected patients (21.0%, 95% CI: 9.9–35.0%) than that among treated patients (16.1%, 95% CI: 3.9–34.3%). The differences were statistically significant for all subgroups except publication language (*p* = 0.13). Substantial heterogeneity was present within all subgroups except for HIV voluntary counseling and testing (VCT) clinics, and unknown HIV transmission route subgroups (Table 2; Supplementary Figure 1).

### Meta regression analysis

The results from our multivariable meta-regression indicated that recruitment site (STD clinic: coefficient = 0.49, *p* < 0.001) and study region (northwestern China: coefficient = −0.18, *p* = 0.03) contributed to the highest heterogeneity across studies (Table 3).

**Table 3:** Raw coefficients from the multivariable meta-regression.

**Predictor**	**Studies, No**.	**Participants, No**.	**Coefficient**	***p*-values**
**Sample size**				
< 600	14	6,477	Ref	Not applicable
**≥**600	14	25,949	−0.05	0.28
**Publication language**				
Chinese	21	26,945	Ref	Not applicable
English	7	5,481	−0.07	0.22
**Study quality**				
Moderate	14	10,831	Ref	Not applicable
High	14	21,595	0.04	0.54
**Recruitment site**				
Local CDC	10	17,541	Ref	Not applicable
Infectious disease hospital	9	9,502	0.09	0.15
VCT clinic	2	1,060	−0.03	0.70
STD clinic	2	1,013	0.49	< 0.001
Community	5	3,310	0.11	0.25
**Study region**				
Northeast	4	3,751	Ref	Not applicable
North	5	6,124	−0.04	0.65
Northwest	3	1,595	−0.18	0.03
East	8	9,319	−0.08	0.28
Central-south	3	6,627	−0.13	0.12
Southwest	5	5,010	−0.14	0.10

CDC, Center for Disease Control and Prevention; STD, Sexually transmitted disease; VCT, Voluntary counseling and testing.

### Publication bias

**Figure 4:**
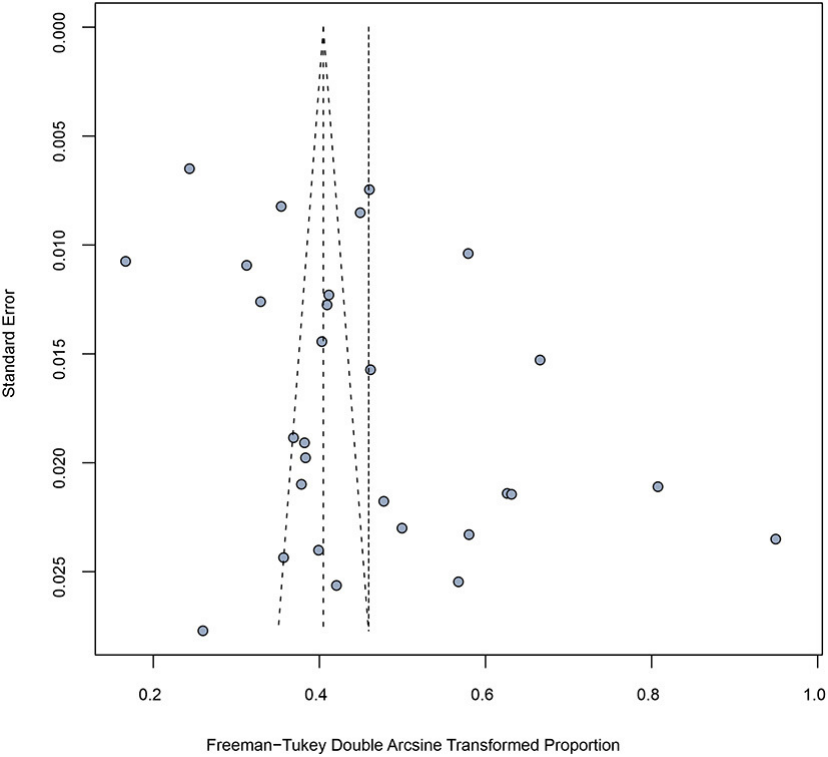
Funnel plot of included studies showing estimate of syphilis prevalence among people living with HIV.

The funnel plot (Figure 4) and Egger's test were similarly not suggestive of publication bias. When subgroup analyses were conducted, publication bias was found for the following grouping variables: study region, age, and CD4 + T cell count (Table 2).

### Sensitivity analysis

In the sensitivity analysis (Supplementary Table 5), when removing a study (34) that focused on middle-aged and elderly people from an STD clinic whose prevalence was an outlier, the pooled prevalence of syphilis among PLWH remained high (18.6%, 95% CI: 14.8–22.6%). The results of the leave-one-out method showed that none of the studies had a disproportionate effect on the pooled estimate (Supplementary Table 6).

## Discussion

This meta-analysis from 29 eligible studies involving 34,740 individuals found that the overall prevalence of syphilis among PLWH in China reached 19.9% (95% CI: 15.4–24.8%), which was higher than the prevalence of syphilis among other populations, such as MSM (10.9%) (51) and drug users (7.8%) (52). The findings demonstrated that PLWH could be as an important high-risk group for syphilis and the urgent need for enhanced syphilis monitoring and management programs in this population.

The estimated syphilis prevalence among PLWH in China was similar to Brazil (20.5%) (53), and higher than Singapore (12.3%) (54) and Turkey (8%) (55). Our estimate was approximately twice as high as that reported in a systematic review published by Kalichman et al. in 2011 (9.5%) (56), and there may be two potential reasons for the difference. First, differences in study time, Kalichman et al. conducted their systematic review ~10 years ago. Second, differences in study regions and populations, of the 37 studies included in this article, 34 (92%) were conducted in non-Asia regions, mainly in North America and Latin America/Caribbean, however, our study only focused on the Chinese mainland population, thus the difference may be attributable to variation of geographical and social environment as well as ethnicity.

The results of subgroup analyses showed higher prevalence of syphilis in the following groups: MSM, STD clinic attendees, and males. In this study, more than half of the STD clinic attendees and male participants were MSM. MSM are at a higher risk of HIV and syphilis infection because of risk behaviors such as multiple sexual partners and unprotected anal intercourse (57). A meta-analysis reported that the prevalence of HIV and syphilis among MSM in China was 7.7 and 10.9% in 2010–2013, respectively (51). This study showed that the prevalence of syphilis among MSM living with HIV in China was 21.9%. Public Health England reported that the prevalence of syphilis among MSM living with HIV in England was 14.1 to 17.0% from 2014 to 2019, lower than our estimate, and MSM who were HIV-positive had over 5 times the rate of syphilis than those who were HIV-negative or of unknown HIV status (58). In China, the national guidelines for HIV screening require HIV testing among all patients with syphilis and other sexually transmitted infections (STIs) (59), but syphilis screening among PLWH has not been clearly included in any guidelines. To efficiently control and prevent both HIV and syphilis, it is necessary to consider PLWH be one of the high priority populations for syphilis monitoring and management, and strengthen syphilis screening among PLWH, especially among sex active MSM living with HIV, to whom syphilis screening every 3 months should be recommended (58). Studies from other countries have reported that “chemsex” (60) and “seroadaptive” (61) behaviors in this group accelerated syphilis transmission among MSM, but we are lacking such data in China, suggesting that future research needs to be carried out in this area.

In the subgroup analysis of CD4 + T cell count, we found that the lower the CD4 + T cell count, the higher the prevalence of syphilis among PLWH. This is consistent with the finding from a study conducted in Canada that coinfection with syphilis can exacerbate the progression of HIV (62). On the other hand, HIV infection also has an effect on syphilis that immunocompromised individuals are less able to mount a protective response against *T. pallidum* (56), increasing the chance of progressing to neurosyphilis, ocular syphilis, and otosyphilis. Subgroup analysis by ART status showed lower syphilis prevalence among treated HIV-infected patients than among non-treated patients, suggesting that ART may reduce susceptibility to syphilis among PLWH. China has implemented pre-exposure prophylaxis (PrEP) as an intervention for HIV infection (63), but its impact on syphilis and other STIs is unclear.

The present study revealed significant heterogeneity across the included 29 studies, we found that the recruitment site and study region were the sources of study heterogeneity through multivariable meta-regression analyses. The pooled syphilis prevalence among PLWH attending STD clinics (59.2%) was significantly higher than that of participants in other recruitment sites, and the prevalence of syphilis in northern China was twice as high as that in northwestern China. It is easy to understand that there are differences in some characteristics of the included participants in different recruitment sites and regions, such as variations in sexual behaviors and social culture in the large country (64, 65).

In summary, we think that it is urgent to develop national guidelines on the screening, monitoring and management of syphilis among PLWH, furthermore implementing protocols suitable to variations for sexual behaviors and social culture in different regions and subgroups. The China's Plan for Syphilis Prevention and Control (2010–2020) called for syphilis prevalence monitoring to be combined with local AIDS monitoring efforts (66). This requires the close collaboration between the National Center for HIV/AIDS Control and Prevention (NCAIDS) and the National Center for STD Control and Prevention (NCSTD) to develop integrated national guidelines for screening, monitoring, and management of syphilis and HIV, under the new national action plans for syphilis/HIV control in future, in order to find out the potential syphilis and HIV coinfection and combat the double epidemics.

In addition, the PLWH are required to receive treatment at designated hospitals in China. Based on the current estimate of 1.05 million PLWH by the Chinese CDC (8) and the 19.9% overall prevalence of syphilis among PLWH from this meta-analysis, we estimated that about 209,005 PLWH are co-infected with syphilis and need medical care for syphilis. Because at those designated HIV treatment hospitals, doctors are not sufficiently trained in diagnosis and treatment of syphilis (67), we recommend developing syphilis training programs tailored to the needs of these HIV physicians, reducing the burden of syphilis and preventing syphilis patients with HIV from progressing to malignant syphilis.

## Strengths and limitations

To our knowledge, this is the first study to systematically review the prevalence of syphilis among PLWH in China. We conducted meta-analysis to summarize prevalence estimates and identify factors associated with syphilis infection among PLWH. We tried to include high-quality articles to get more accurate and reliable results.

However, there are some limitations to the present meta-analysis. Firstly, there was significant heterogeneity across the included studies. We conducted subgroup analyses and meta-regression analyses to investigate the source of heterogeneity, 11 relevant variables can be used for subgroup analyses, but only five of them can be employed for multivariable meta-regression analyses, and found that recruitment site and study region induced substantial heterogeneity, other six variables that were not included in the meta-regression analyses due to the variation in study design and data collection across these studies, may not be still excluded as the source of heterogeneity. Secondly, although FSWs have been identified as a target population for syphilis and HIV control and prevention (3), none of the published literatures searched include study on syphilis prevalence in FSWs living with HIV, we cannot estimate the pooled syphilis prevalence among this subpopulation, this may be due to very low HIV prevalence in FSWs in China (0.19%) (68). Thirdly, publication bias was present in three subgroup analyses, including study region, age, and CD4 + T cell count, and the results of such subgroup analyses should be interpreted with caution.

## Conclusion

In conclusion, we found that the prevalence of syphilis among PLWH was markedly high in China, especially among MSM living with HIV. Findings from this meta-analysis suggested that PLWH should be one of the priority populations for syphilis control and prevention, and the strategies include regular syphilis testing and consistent condom use. Furthermore, developing national guidelines for the integrated screening, monitoring, and management of HIV and syphilis as well as syphilis diagnosis and treatment training programs for physicians at designated HIV treatment hospitals is urgent and crucial to combat HIV and syphilis coinfection in China.

## Data availability statement

The original contributions presented in the study are included in the article/Supplementary material, further inquiries can be directed to the corresponding author.

## Author contributions

XG conceived and designed the study. YW, WZ, and XG carried out the literature searches, extracted the data, and assessed the study quality. YW, WZ, XY, CS, and MZ performed the statistical analysis. YW and WZ wrote the manuscript. XG and GF revised the manuscript. All authors contributed to the article and approved the submitted version.

## Funding

This work was supported by Guizhou Provincial Project of Science and Technology (Grant Number: 2021-026). The funder had no role in study design, data collection, analysis and interpretation of data, writing of the manuscript, and the decision to submit the manuscript for publication.

## Conflict of interest

The authors declare that the research was conducted in the absence of any commercial or financial relationships that could be construed as a potential conflict of interest. The reviewer LS declared a shared affiliation with one of the authors GF to the handling editor at the time of review.

## Publisher's note

All claims expressed in this article are solely those of the authors and do not necessarily represent those of their affiliated organizations, or those of the publisher, the editors and the reviewers. Any product that may be evaluated in this article, or claim that may be made by its manufacturer, is not guaranteed or endorsed by the publisher.
