# Supplementary material for: Prevalence of syphilis among people living with HIV and its implication for enhanced coinfection monitoring and management in China: A meta-analysis
Source: Front Public Health. 2022 Oct 17;10:1002342. doi: 10.3389/fpubh.2022.1002342 (PMC9618949; doi:10.3389/fpubh.2022.1002342)
Supplement: Supplementary file 1 [file Data_Sheet_1.docx]

Supplementary Material

Supplementary Table 1. Search strategy

| Search database | Search terms |
| --- | --- |
| Medline (Ovid interface) | #1 exp human immunodeficiency virus/ |
|  | #2 (HIV or human immuno-deficiency virus or human immunodeficiency virus$ or AIDS or acquired immune deficiency syndrome).ti,ab. |
|  | #3 #1 or #2 |
|  | #4 exp Syphilis/ |
|  | #5 (syphilis or treponema or treponemal or treponema pallidum).ti,ab. |
|  | #6 #4 or #5 |
|  | #7 exp co-infection/ |
|  | #8 (accumulated infection or Coinfections or Mixed Infection or Infection, Mixed or Infections, Mixed or Mixed Infections or Polymicrobial Infection or Infection, Polymicrobial or infections, Polymicrobial or Polymicrobial Infections or Co-infection or Co infection or Co-infections or Secondary Infections or Infection, Secondary or Infections, Secondary or Secondary Infection).ti,ab. |
|  | #9 #7 or #8 |
|  | #10 (China or Chinese).ti,ab. |
|  | #11 #3 and #6 and #9 and #10 |
| Medline (PubMed interface) | #1 HIV[Mesh] |
|  | #2 HIV[tiab] OR "human immuno-deficiency virus"[tiab] OR "human immunodeficiency virus*"[tiab] OR AIDS[tiab] OR "acquired immune deficiency syndrome"[tiab] |
|  | #3 #1 OR #2 |
|  | #4 Syphilis[Mesh] |
|  | #5 syphilis[tiab] OR treponema[tiab] OR treponemal[tiab] OR "treponema pallidum"[tiab] |
|  | #6 #4 OR #5 |
|  | #7 co-infection[Mesh] |
|  | #8 Coinfections[tiab] OR "Mixed Infection"[tiab] OR "Infection, Mixed"[tiab] OR "Infections, Mixed"[tiab] OR "Mixed Infections"[tiab] OR "Polymicrobial Infection"[tiab] OR "Infection, Polymicrobial"[tiab] OR "infections, Polymicrobial"[tiab] OR "Polymicrobial Infections"[tiab] OR Co-infection[tiab] OR "Co infection"[tiab] OR Co-infections[tiab] OR "Secondary Infections"[tiab] OR "Infection, Secondary"[tiab] OR "Infections, Secondary"[tiab] OR "Secondary Infection"[tiab]) |
|  | #9 #7 OR #8 |
|  | #10 China[tiab] OR Chinese[tiab] |
|  | #11 #3 and #6 and #9 and #10 |
| Embase | #1 'hiv'/exp OR 'aids'/exp |
|  | #2 hiv:ti,ab OR 'human immuno-deficiency virus':ti,ab OR 'human immunodeficiency virus?':ti,ab OR aids:ti,ab OR 'acquired immune deficiency syndrome':ti,ab |
|  | #3 #1 or #2 |
|  | #4 'syphilis'/exp |
|  | #5 syphilis:ti,ab OR treponema:ti,ab OR treponemal:ti,ab OR 'treponema pallidum':ti,ab |
|  | #6 #4 OR #5 |
|  | #7 'co infection'/exp |
|  | #8 coinfections:ti,ab OR 'mixed infection':ti,ab OR 'infection, mixed':ti,ab OR 'infections, mixed':ti,ab OR 'mixed infections':ti,ab OR 'polymicrobial infection':ti,ab OR 'infection, polymicrobial':ti,ab OR 'infections, polymicrobial':ti,ab OR 'polymicrobial infections':ti,ab OR 'co infection':ti,ab OR 'co infections':ti,ab OR 'secondary infections':ti,ab OR 'infection, secondary':ti,ab OR 'infections, secondary':ti,ab OR 'secondary infection':ti,ab |
|  | #9 #7 OR #8 |
|  | #10 china:ti,ab OR chinese:ti,ab |
|  | #11 #3 and #6 and #9 and #10 AND [1990-2022]/py |

Supplementary Table 2. Details of excluded studies

| **Primary reason for exclusion** | **First author** | **Published year** | **Title** |
| --- | --- | --- | --- |
| HIV and syphilis infections were not confirmed by standard laboratory serologic testing | Luo JL | 2003 | The status of HCV, HBV and HGV infection among people living with HIV |
|  | Gu YC | 2004 | HIV and syphilis infection and related risk behavior among drug users in Zhongshan, 1999-2003 |
|  | Gu YC | 2005 | Investigation of AIDS and syphilis infection in drug abusers and their knowledge and behaviors related to the diseases |
|  | Han L | 2005 | Analysis of blood test results among voluntary blood donors in Nanchang city |
|  | Liu J | 2005 | Analysis of results of blood test for 11295 blood donation volunteers |
|  | Jiang HY | 2006 | Investigation and analysis of hepatitis B, hepatitis C, syphilis and HIV infection among women serving in entertainment establishments |
|  | Li XJ | 2006 | Analysis of the results from sentinel surveillance of HIV infection in Hefei city |
|  | Liu G | 2006 | Analysis on HIV compostive surveillance in high risk groups in Sichuan |
|  | Song HQ | 2007 | Comparison of High Risk Behaviors, the Prevalence of HIV、HCV and Syphilis among Drug Users Enrolled through Different Recruitment Methods in Xichang County of Sichuan Province, China |
|  | Song SZ | 2007 | Survey of syphilis and HIV infection among food service workers in Ganjingzi District, Dalian City from 1997 to 2005 |
|  | Wang AX | 2007 | Investigation on the syphilis infection of HIV carriers in a certain area of southern Henan Province |
|  | Cui YL | 2008 | Investigation of HIV, syphilis and hepatitis infection status in high-risk population |
|  | Li XW | 2008 | Surveillance and analysis of infectious diseases among drug users in Longyan City |
|  | Wang AX | 2008 | Investigation and analysis of HCV, HBV and syphilis co-infection in HIV-infected persons |
|  | Ouyang L | 2009 | A respondent-driven sampling survey on HIV and risk factors among men who have sex with men in Chongqing |
|  | Qi MC | 2009 | Effect of needle sharing on the prevalence of hepatitis C, hepatitis B, and syphilis infection in HIV-infected patients |
|  | Liu RX | 2010 | Analysis of HIV positivity and co-infection status in blood donation specimens |
|  | Ke WJ | 2011 | A survey of mixed infection with pathogens in sexually transmitted disease clinic |
|  | Qiu W | 2011 | Analysis of the results of 3926 cases of pre-transfusion infectious index testing |
|  | Wang R | 2011 | Epidemiological characteristics of HIV/AIDS cases |
|  | Chen D | 2012 | Research on the situation of HIV/AIDS coinfected with HBV，HCV，TP and other diseases in Wenzhou area |
|  | He J | 2012 | Analysis of present situation of genital herpes patients combined with TP, HIV and HCV infections |
|  | Qi XT | 2012 | Analysis of co-infection with pathogenic microorganisms in clinical AIDS patients |
|  | Yuan H | 2012 | Relationship between HBV infection and HIV, HCV and TP infection in certain area |
|  | Zhao YS | 2012 | Seroprevalence of hepatitis C, hepatitis B virus and syphilis in HIV-1 infected patients in Shandong, China |
|  | Zhu B | 2012 | Research on effect of AIDS coinfection with HBV，HCV and TP on CD4 |
|  | Wang J | 2012 | HIV, syphilis and HSV-2 prevalence in relation to receptive, insertive and both sex roles among MSM in eight cities of China |
|  | Duan J | 2013 | Evaluation of AIDS prevention with risk-reducing measures among drug abusers in Gucheng District, Lijiang City, Yunan Province |
|  | Tang YF | 2013 | Co-infection of HIV and HBV, HCV, TP in Liangshan |
|  | Yang MY | 2014 | Analysis on test results of infected HIV-1 merger syphilis and hepatitis C in Northeast area of Chongqing |
|  | Liu YZ | 2015 | Analysis of co-infections among blood donation volunteers |
|  | Wei GL | 2015 | Study on the changing trend of HIV, HCV and TP infection in patients with methadone replacement therapy |
|  | Ao YY | 2016 | Analysis of HIV, HCV and syphilis infections in patients undergoing methadone maintenance treatment in Yangjiang City |
|  | Pu YF | 2016 | Analysis of syphilis, HIV, HCV and HBV infection among blood donation volunteers in Changzhou City from 2011 to 2014 |
|  | Cao W | 2017 | Prevalence of human immunodeficiency virus, hepatitis B virus, hepatitis C virus and treponema pallidum infections among patients before blood transfusion in Xiangya Hospital Central South University, China from 2011 to 2015 |
|  | Chen RL | 2017 | Analysis of the detection results of HIV and syphilis in HIV voluntary counseling in Futian district of Shenzhen from 2010-2016 |
|  | Li YB | 2017 | Epidemiological characteristics of HIV/AIDS combined with syphilis infection |
|  | Lv JE | 2017 | HIV merger TP/HCV/HBV infection results analysis |
|  | Xu L | 2017 | Prevalence of HIV/HBV,HIV/HCV and HIV/syphilis co-infection |
|  | Cao WW | 2018 | Prevalence of hepatitis B virus, hepatitis C virus, human immunodeficiency virus and Treponema pallidum infections in hospitalized patients before transfusion in Xiangya hospital Central South University, China from 2011 to 2016 |
|  | Chen JT | 2018 | Situtation of HIV patients co-infected with TP, HBV and HCV in Maoming city and its relationship with CD4+T lymphocytes |
|  | Shi CH | 2018 | Analysis of new AIDS epidemic and hepatitis B, hepatitis C and syphilis co-infection in Pingdingshan City |
|  | Xu WJ | 2018 | HBV, HCV and syphilis infection among HIV-positive people in Nanjing from 2015 to 2016 |
|  | Yang RR | 2018 | The situation of sexual transmitted diseases among acquired immune deficiency syndrome patients and its influence on the risk of human immunodeficiency virus transmission between couples |
|  | Yang T | 2018 | High prevalence of syphilis, HBV, and HCV co-infection, and low rate of effective vaccination against hepatitis B in HIV-infected patients in West China hospital |
|  | Zhang C | 2018 | Analysis of 1018 HIV/AIDS patients co-infection with HBV, HCV, and TP in Shaanxi |
|  | Bi C | 2019 | Analysis on the pathogens of sexually transmitted diseases among women |
|  | Chen JQ | 2019 | Analysis of characteristics of 1870 HIV/AIDS patients co-infected with HBV, HCV and TP in Jiangxi Province |
|  | Chen YL | 2019 | Analysis of HIV, syphilis and HCV multi -virus infection status in AIDS voluntary counseling and testing population in Futian District, Shenzh |
|  | Feng QX | 2019 | A retrospective survey on TP and HIV infection status of voluntary blood donors in Qingdao from 2011-2017 |
|  | Shi CH | 2018 | Analysis of new AIDS epidemic and hepatitis B, hepatitis C and syphilis co-infection in Pingdingshan City |
|  | Xu WJ | 2018 | HBV, HCV and syphilis infection among HIV-positive people in Nanjing from 2015 to 2016 |
|  | Ding JP | 2020 | Expression level and clinical significance of immune cells in peripheral blood of 347 HIV/AIDS patient |
|  | Guo JX | 2020 | Analysis on merged positive infection situation of contagious serum markers in voluntary blood donors in Nanping City from 2011 to 2018 |
|  | He JS | 2020 | Analysis on the status and characteristics of HIV co-infection with hepatitis B, hepatitis C and syphilis in voluntary blood donors |
|  | Huang L | 2020 | Syphilis infection does not affect immunodeficiency progression in HIV-infected men who have sex with men in China |
|  | Lan LY | 2020 | Co-infection status of HIV with HBV, HCV and TP in unpaid blood donors |
|  | Lv FY | 2020 | HIV and syphilis infection in blood donors in Dalian during 2017-2019 |
|  | Yang Q | 2020 | Analysis of the current situation of co-infection of HIV with HBV, HCV and TP among Yi nationality in a county of Liangshan Prefecture |
|  | Zhang C | 2020 | Epidemiological features and risk factors for acquiring hepatitis b, hepatitis c, and syphilis in HIV-infected patients in Shaanxi province, northwest China |
|  | Zhang GD | 2020 | Detection and study of hepatitis B virus, hepatitis C virus and syphilis in AIDS patients |
|  | Mamatiaili Wubuli | 2020 | Analysis about the Characteristics of HCV, HBV and TP Infection in 381 AIDS patients |
|  | Huang S | 2021 | Analysis of the characteristics of HIV/AIDS patients co-infected with pathogenic microorganisms in Yunnan from 2015 to 2019 |
|  | Zhang L | 2021 | Research on the results of HIV infection and HBV, HCV, TP and TB testing study |
| HIV-infected participants < 300 | Du JW | 2001 | Evaluation on Effects of AIDS Intervention among Drug Users in Hainan Province |
|  | Jiang AP | 2003 | Analysis of high-risk female infected with sexually transmitted diseases in Fuzhou area |
|  | Luo XM | 2003 | Serological analysis of infection markers in special population |
|  | Lv GX | 2005 | A study on HIV, TB, HBV and syphilis infection in heroin addicts |
|  | Zhang Y | 2005 | Analysis of anti-HCV, anti-HIV and syphilis co-infection in blood donors |
|  | Chen B | 2006 | Analysis of HBV, HCV and TP infection among Anti-HIV positive blood donors in Wuxi city |
|  | Wei XS | 2006 | Analysis of HBV, HCV, syphilis and tuberculosis infection status among anti-HIV positive patients |
|  | Zhu JD | 2006 | Survey on HIV, HBV and syphilis infection in drug users in Ningbo |
|  | Li Y | 2009 | Analysis of HIV and syphilis surveillance results of entry-exit personnel at Suifenhe Railway Station |
|  | Liang L | 2009 | Investigation and analysis of HIV infection among men who have sex with men |
|  | Hong J | 2010 | Infection State of 4 Kinds of Common Blood-borne Pathogens in Hubei Province |
|  | Liu JB | 2010 | Study of HIV, HBV, HCV and syphilis cross-infection in pre-surgical and pre-transfusion patients |
|  | Wang LL | 2010 | Application of respondent-driven sampling method in HIV surveillance among men who have sex with men |
|  | Guan L | 2011 | Analysis of HIV-positive blood donors co-infected with HBV, HCV and TP |
|  | Jiang ZX | 2011 | Infection situations of HIV and syphilis of 1300 drug abusers in Qingdao city and analysis of related fators |
|  | Qiu XM | 2011 | Epidemiological analysis of 822 cases of sexually transmitted diseases in high risk group in Hangzhou City |
|  | Li GQ | 2012 | Analysis on the infection situtation of AIDS, hepatitis C and syphilis of Shenzhen Longgang district drug abusers |
|  | Wu HL | 2012 | Analysis of syphilis and HIV co-infection in STD patients in Quanzhou |
|  | Cai YM | 2013 | Factors associated with syphilis infection among 2943 men who had sex with men in Shenzhen |
|  | Liu SJ | 2013 | Survey of HIV and syphilis infection among 450 men who have sex with men in Shijiazhuang |
|  | Jiang YQ | 2014 | Monitoring results analysis of HIV, syphilis and HCV in national levels drug monitoring sentinel Xixiu district in Anshun city during 2010-2013 |
|  | Wang GL | 2014 | Analysis of the effect of high-risk behavior intervention for female sex workers in entertainment venues in Suzhou City |
|  | Xiao MM | 2014 | Studies on coinfection and leucocyte counts in HIV infection |
|  | Wang XF | 2014 | HIV and syphilis prevalence trends among men who have sex with men in Guangxi, China: yearly cross-sectional surveys, 2008-2012 |
|  | Xu Q | 2015 | Analysis of HIV and syphilis test results among MSM in Yangzhou in 2014 |
|  | Yang Y | 2015 | Analysis on HIV and syphilis infections among attendants of HIV voluntary counseling and testing in Xi'an City |
|  | Qin SQ | 2016 | Effect of four kinds bloodborne pathogens on human serum neopterin level |
|  | Wang H | 2016 | Analysis of AIDS sentinel surveillance results of drug users in Tongren City from 2001 to 2015 |
|  | Ma L | 2016 | Analysis of HIV/syphilis infection status and influencing factors of syphilis infection in VCT clinics in Guiyang |
|  | Dai W | 2017 | Prevalence of HIV and syphilis co-infection and associated factors among non-commercial men who have sex with men attending a sexually transmitted disease clinic in Shenzhen, China |
|  | Liu Y | 2017 | Co-infections of HIV, syphilis and HSV-2 among men who have sex with men at the voluntary HIV counseling and testing clinks in Shanghai |
|  | Wang Y | 2018 | Prevalence of syphilis and related factors in men who have sex with men in Mianyang, Sichuan |
|  | Zhao YH | 2018 | Infection of Treponema pallidum in HIV high-risk population in Miyun District of Beijing from 2008-2017 |
|  | Shi WY | 2019 | HIV and syphilis infection status and epidemiological characteristics of patients in a STD clinic in Beijing from 2013 to 2017 |
|  | Ma XJ | 2021 | Analysis of the detection results of human immunodeficiency virus, syphilis and hepatitis C among men who have sex with men in Yangzhou from 2014 to 2019 |
| No required data | Pu DM | 2004 | Epidemiological analysis of sexually transmitted diseases in outpatient clinics of Tongji Hospital from 1987 to 2001 |
|  | Lv SC | 2006 | Analysis of the epidemiological situation of infectious diseases in Chongzhou City of Sichuan Province from 1950 to 2005 |
|  | Yang SY | 2006 | An Analysis of 22 084 Cases of STDs in Tianjin Medical University General Hospital |
|  | Chen FP | 2009 | Analysis of syphilis infection among employees in public places in Changxing in 2009 |
|  | Pao YG | 2009 | HIV infection and KAP status among men who have sex with men in 14 Chinese cities |
|  | Chang ZR | 2010 | A survey of HIV, HCV, syphilis and HSV-2 among drug users attending methadone maintenance treatment clinics in Guangxi and Guizhou |
|  | Dong JG | 2010 | Survey of hepatitis C, syphilis, AIDS and hepatitis B among drug users in Qinhuai District of Nanjing from 2009 to 2010 |
|  | Zhang YH | 2010 | Analysis of HIV/Syphilis/HCV infection among drug users in 15 cities’ China |
|  | Pu AH | 2011 | Analysis of the results of HIV/AIDS surveillance among drug users in Jianshui County from 2007 to 2009 |
|  | Kang JH | 2012 | Study on the co-infection of HBV, HCV and syphilis among HIV-infected patients in Xiangyang City |
|  | Yang FQ | 2012 | Study on the co-infection of HBV, HCV and syphilis among HIV-infected patients in Xiangyang City |
|  | Nong SY | 2013 | HIV and syphilis screening among hospitalized patients in the general hospital |
|  | Wang P | 2013 | The infection of hepatitis C virus and treponema pallidum among HIV single-infection couples in Queshan county |
|  | Zhu QY | 2013 | Cross-sectional survey of male HIV-positive drug users in Guangxi |
|  | Ba DM | 2014 | The clinical manifestation and treatment of syphilis and HIV co-infection in Xinjiang |
|  | Liang L | 2014 | Infection status of HIV and its influence factors among men who havesex with men in Sichuan province |
|  | Qin CW | 2014 | Characteristics of high risk behaviors and condition of HIV/Syphilis infection among female sex workers at deferent grades in Guigang |
|  | Shan D | 2014 | A study on the effectiveness of prevention of mother to child HIV, syphilis, and hepatitis B among pregnant women in Dehong prefecture, Yunnan province, China from 2011 to 2013 |
|  | Xu J | 2014 | Clinical application study of transfusion-associated infectious disease index testing before blood transfusion |
|  | Ma ZM | 2015 | Investigation on AIDS knowledge, belief and practice among female sex workers in public places in Qilian County |
|  | Su MH | 2015 | Analysis of serological testing for HIV and syphilis among blood donors in Jiamusi |
|  | Tong Y | 2015 | Study on the co-infection of TP among HIV-infected patients in Guiyang City |
|  | Jia Z | 2015 | HIV burden in men who have sex with men: a prospective cohort study 2007-2012 |
|  | Guan LQ | 2016 | Spectrum of central nervous system disorders in the first hospitalized HIV/AIDS patients |
|  | Ma WT | 2017 | Discussion on providing follow-up services to syphilis screen-positive voluntary blood donors |
|  | Song X | 2018 | Basic characteristics and HIV／syphilis infection status of the clients to the AIDS voluntary counseling and testing clinic in Qingdao from 2012 to 2015 |
|  | Jiang HH | 2019 | Analysis of AIDS sentinel surveillance results among drug users in Chongqing from 2010 to 2017 |
|  | Song LH | 2019 | Analysis of the clients to HIV/AIDS voluntary counseling and testing clinics in Tianshan district of Urumqi |
|  | Tang Y | 2019 | Prevalence of HIV, hepatitis C and syphilis infections based on sentinel surveillance during 2013-2017 in Pudong new district of Shanghai |
|  | Song BL | 2020 | HIV infection and risk factors among community drug users in Xichang city, Sichuan province from 2009 to 2016 |
|  | Song LJ | 2020 | Analysis of AIDS sentinel surveillance among pregnant women in Yunnan from 2010 to 2018 |
|  | Zhang XH | 2020 | Analysis of the HIV antibody screening results among the preoperative examination patients in Beijing Tongren Hospital from 2008-2018 |
|  | Wang WH | 2021 | Prevalence of HIV patients co-infected with HBV, HCV and TP in the general hospital |
| No description of HIV/syphilis testing method | Zhen J | 2010 | HIV Sentinel Surveillance in a Detoxification Center in Hengyang, Hunan Province During the Period of 2002~2008 |
|  | Zhou C | 2011 | Study on the prevalence and assciated factors of HIV and syphilis among 1166 men who sex with men |
|  | Chang YH | 2014 | Associated factors with syphilis among human immunodeficiency virus-infected men who have sex with men in Taiwan in the era of combination antiretroviral therapy |
|  | Pan YJ | 2014 | Analysis of Co-infection with other pathogens in HIV/ AIDS patients |
|  | Che XW | 2015 | An analysis of HIV infection situation and its influencing factors among VCT population in Taiyuan City |
|  | Chen YC | 2015 | The rising trend of sexually transmitted infections among HIV-infected persons: A population-based cohort study in taiwan, 2000 through 2010 |
|  | Lu TY | 2015 | Analysis of the results of HIV-positive co-infection with syphilis and hepatitis C in different people sent for testing |
|  | Pan H | 2015 | To Study the Molecular Epidemiology and Antiviral Therapy of the Human Immunodeficiency Virus CRF01-AE Subtype in Xinyu City |
|  | Wu QM | 2015 | HIV infection and syphilis prevalence among men who have sex with men receiving voluntary counseling and testing appointed through a web-based registering system and related factors |
|  | Feng J | 2016 | Analysis on merged positive infection situation of contagious serum markers in voluntary blood donors |
|  | Lu TY | 2016 | The overlapping infection status and the related research of syphilis and hepatitis C virus with AIDS in Qiannan areas |
|  | Su XF | 2016 | Analysis of HIV voluntary counseling and testing among people aged above 50 in Yunan province from 2011-2014 |
|  | Ye Yekejiergeli | 2016 | HIV, HCV, syphilis and their co-infection and risk factors analysis among drug users in Urumqi from 2010-2014 |
|  | Liang PP | 2017 | Clinical epidemiology of 359 cases of acquired immunodeficiency syndrome and pulmonary tuberculosis co-infection |
|  | Yang GY | 2017 | Analysis and prevention strategies of HIV infection among voluntary blood donors in Taizhou |
|  | Gao JH | 2018 | Analysis on sociological characteristics of double infection of HIV and mycobacterium tuberculosis ,Baise city |
|  | Li SJ | 2018 | Analysis on the Status of HIV Infection of Voluntary Non-remunerated Blood Donors in Guangzhou Area, 2010-2015 |
|  | Li XX | 2018 | HIV infection and high-risk behavior characteristics among MSM population in Longgang district, Shenzhen city |
|  | Ma KH | 2019 | Infection characteristics of HIV infected patients in Dazhou between 2014 to 2017 |
|  | Wang YQ | 2020 | Characteristics of AIDS epidemic and opportunistic infections in Weifang City |
|  | Jin LH | 2022 | Analysis of syphilis infection in newly diagnosed HIV/AIDS patients in Jinhua from 2017 to 2020 |
| Same study sample | Cai YM | 2012 | Distribution characteristics and factors associated with HIV infection among MSM in Shenzhen |
|  | Cai YM | 2013 | Analysis on the prevalence trend of syphilis/HIV among men who have sex with men in Shenzhen from 2005 to 2011 |
|  | Cai YM | 2013 | Factors associated with syphilis-HIV co-infection among men who have sex with men in Shenzhen city |
|  | Cai YM | 2016 | Factors associated with commercial sexual behavior among men who have sex with men in Shenzhen, China, in 2011-2015 |
|  | Cai YM | 2016 | Factors associated with multiple anal sexual partners among men who have sex with men in Shenzhen |
|  | Cai YM | 2016 | Drug abuse and syphilis/HIV infection among men who have sex with men in Shenzhen |
|  | Song YJ | 2016 | Marital status and its effect on syphilis/HIV infection among men who have sex with men in Shenzhen |
|  | Xu YY | 2016 | Infection status of HIV in men who have sex with men in Nanjing, 2011-2015 |
|  | Cai YM | 2017 | Factors associated with syphilis/HIV infection among men who have sex with men in Shenzhen,2011-2016 |
|  | Cai YM | 2017 | High risk behaviours and syphilis/HIV infection among men who have sex with men aged 50 years and older in Shenzhen, China |
|  | Yu XH | 2017 | Analysis of the current situation of HIV combined with HBV, HCV and syphilis infection in Ningxia area |
|  | Zhun Z | 2019 | Trends in HIV prevalence and risk behaviours among men who have sex with men from 2013 to 2017 in Nanjing, China: a consecutive cross-sectional survey |
|  | Wu YM | 2020 | HIV antibody reexamination and syphilis infection in Nanjing from 2014 to 2019 |
|  | Li DF | 2021 | HCV and Treponema pallidum infection status in HIV/AIDS cases in Yunnan province, January-June, 2020 |
| No age-specific syphilis prevalence | Ma YM | 2012 | Status of HIV and syphilis infection among men who have sex with men in Henan Province in 2011 |
|  | Yang CK | 2013 | Prevalence and predictors of syphilis and herpes simplex type 2 virus (HSV-2) infections among HIV-infected men who have sex with men in Jiangsu province |
|  | Zhang HL | 2014 | Characteristics of HIV infected MSM and their syphilis infection status in Xi'an |
|  | Chen L | 2016 | Investigation and analysis of hepatitis B, hepatitis C and syphilis infection in HIV-infected patients |
|  | Li L | 2019 | Analysis of the characteristics, HIV and syphilis infection among the clients to a VCT clinic during 2012-2017 |
|  | Xu LP | 2019 | Study on the affect of syphilis infection on CD4^+^ lymphocytes in HIV-infected/AIDS patients in Yunnan Province in 2019 |
| Irrelevant reports | Yang HX | 2008 | Treatment of infectious diseases during pregnancy based on the principles of evidence-based medicine |
|  | Zhong LH | 2008 | Study on the necessity of pre-pregnancy health education for patients with perinatal infection and family members |
| Review | Muessing KE | 2010 | HIV and syphilis among men who have sex with men in China: The time to act is now |

Abbreviation: PLWH, people living with HIV.

Supplementary Table 3. Guidelines for critically appraising studies of prevalence or incidence of a health problem (1)

| **A. ARE THE STUDY METHODS VALID?**  1. Are the study design and sampling method appropriate for the research question?  2. Is the sampling frame appropriate?  3. Is the sample size adequate?  4. Are objective, suitable and standard criteria used for measurement of the health outcome?  5. Is the health outcome measured in an unbiased fashion?  6. Is the response rate adequate? Are the refusers described?  **B. WHAT IS THE INTERPRETATION OF THE RESULTS?**  7. Are the estimates of prevalence or incidence given with confidence intervals and in detail by subgroup, if appropriate?  **C. WHAT IS THE APPLICABILITY OF THE RESULTS?**  8. Are the study subjects and the setting described in detail and similar to those of interest to you? |
| --- |

Supplementary Table 4. Study quality assessment by Loney’s 8-item scale

|  | **Study Design and Sampling Method** | **Sampling Frame** | **Sample Size** | **Appropriate Measurement** | **Unbiased Measurement** | **Response Rate** | **Estimates of prevalence** | **Description of study subjects** | **Quality assessment score** |
| --- | --- | --- | --- | --- | --- | --- | --- | --- | --- |
| Zhang YQ et al (2012) | 0 | 1 | 1 | 1 | 1 | 1 | 1 | 0 | 6 |
| Gao K et al (2013) | 1 | 1 | 1 | 1 | 1 | 1 | 1 | 1 | 8 |
| Wang ZY et al (2013) | 0 | 0 | 1 | 1 | 0 | 1 | 1 | 0 | 4 |
| Wu HB et al (2013) | 1 | 1 | 1 | 1 | 1 | 1 | 1 | 1 | 8 |
| Wu ZY et al (2013) | 1 | 1 | 1 | 1 | 1 | 1 | 0 | 0 | 6 |
| Hu QH et al (2014) | 1 | 1 | 1 | 1 | 0 | 1 | 1 | 1 | 7 |
| Li Y et al (2014) | 1 | 1 | 1 | 1 | 1 | 1 | 1 | 1 | 8 |
| Li JW et al (2016) | 1 | 1 | 1 | 1 | 0 | 1 | 1 | 0 | 6 |
| Ya XR et al (2016) | 1 | 1 | 1 | 1 | 1 | 1 | 1 | 1 | 8 |
| Ma N et al (2016) | 1 | 1 | 1 | 1 | 1 | 1 | 1 | 1 | 8 |
| Cao M et al (2016) | 1 | 1 | 1 | 1 | 0 | 1 | 1 | 1 | 7 |
| Yuan G et al (2017) | 1 | 1 | 1 | 1 | 0 | 1 | 1 | 1 | 7 |
| Wang L et al (2017) | 0 | 0 | 1 | 1 | 1 | 1 | 1 | 1 | 6 |
| Hu QH et al (2017) | 1 | 1 | 1 | 1 | 0 | 1 | 1 | 1 | 7 |
| Liu L et al (2017) | 0 | 0 | 1 | 1 | 1 | 1 | 1 | 0 | 5 |
| Fan SF et al (2017) | 0 | 1 | 1 | 1 | 1 | 1 | 0 | 0 | 5 |
| Zhou QH et al (2018) | 1 | 1 | 1 | 1 | 1 | 1 | 0 | 0 | 6 |
| Liu ZY et al (2018) | 1 | 1 | 1 | 1 | 0 | 1 | 1 | 1 | 7 |
| Zhao H et al (2018) | 1 | 1 | 1 | 1 | 1 | 1 | 1 | 1 | 8 |
| Wu YM et al (2019) | 1 | 1 | 1 | 1 | 1 | 1 | 1 | 1 | 8 |
| Weng RX et al(2019) | 1 | 1 | 1 | 1 | 1 | 1 | 1 | 0 | 7 |
| Gao J et al (2019) | 0 | 0 | 1 | 1 | 1 | 1 | 1 | 0 | 5 |
| Zhu XJ et al (2020) | 0 | 0 | 1 | 1 | 0 | 1 | 1 | 1 | 5 |
| Wang LR et al (2020) | 0 | 1 | 1 | 1 | 1 | 1 | 0 | 0 | 5 |
| Li SF et al (2020) | 0 | 0 | 1 | 1 | 0 | 1 | 1 | 1 | 5 |
| Sun LQ et al (2020) | 1 | 1 | 1 | 1 | 1 | 1 | 1 | 1 | 8 |
| Zhang L et al (2021) | 0 | 0 | 1 | 1 | 0 | 1 | 1 | 0 | 4 |
| Cheng J et al (2021) | 0 | 0 | 1 | 1 | 0 | 1 | 1 | 1 | 5 |
| Tu W et al (2022) | 0 | 0 | 1 | 1 | 1 | 1 | 1 | 0 | 5 |

Supplementary Table 5. Sensitivity analyses with restricted studies in specific conditions

|  | **Number of studies** | **Prevalence, % (95% CI)** | **I^2^ (%)** | **p-value** |
| --- | --- | --- | --- | --- |
| All studies | 29 | 19.9 (15.4-24.8) | 98.9 | <0.001 |
| Excluding studies with extreme syphilis prevalence | 28 | 18.6 (14.8-22.6) | 98.7 | <0.001 |

Supplementary Table 6. Sensitivity analysis with the leave-one-out method

| **Omitted study or studies** | **Prevalence, % (95% CI)** | **I^2^, %** |
| --- | --- | --- |
| Zhang YQ et al, 2012 | 19.8 (15.2-24.9) | 98.9 |
| Gao K et al, 2013 | 20.3 (15.7-25.4) | 98.9 |
| Wang ZY et al, 2013 | 20.2 (15.5-25.3) | 98.9 |
| Wu HB et al, 2013 | 19.6 (15.0-24.6) | 98.9 |
| Wu ZY et al, 2013 | 19.6 (15.0-24.6) | 98.8 |
| Hu QH et al, 2014 | 19.9 (15.3-25.0) | 98.9 |
| Li Y et al, 2014 | 20.2 (15.6-25.3) | 98.9 |
| Li JW et al, 2016 | 19.3 (14.9-24.2) | 98.8 |
| Ya XR et al, 2016 | 20.2 (15.6-25.3) | 98.9 |
| Ma N et al, 2016 | 20.1 (15.4-25.2) | 98.9 |
| Cao M et al, 2016 | 20.2 (15.5-25.3) | 98.9 |
| Yuan G et al, 2017 | 20.4 (15.7-25.4) | 98.9 |
| Wang L et al, 2017 | 18.6 (14.8-22.6) | 98.7 |
| Hu QH et al, 2017 | 19.5 (14.9-24.4) | 98.9 |
| Liu L et al, 2017 | 20.0 (15.4-25.2) | 98.9 |
| Fan SF et al, 2017 | 20.1 (15.4-25.2) | 98.9 |
| Zhou QH et al, 2018 | 20.1 (15.4-25.2) | 98.9 |
| Liu ZY et al, 2018 | 19.6 (15.0-24.7) | 98.9 |
| Zhao H et al, 2018 | 20.2 (15.5-25.3) | 98.9 |
| Wu YM et al, 2019 | 20.0 (15.3-25.1) | 98.9 |
| Weng RX et al, 2019 | 18.9 (14.8-23.5) | 98.8 |
| Gao J et al, 2019 | 20.1 (15.5-25.3) | 98.9 |
| Zhu XJ et al, 2020 | 20.1 (15.4-25.2) | 98.9 |
| Wang LR et al, 2020 | 20.5 (15.9-25.5) | 98.9 |
| Li SF et al, 2020 | 20.8 (16.3-25.6) | 98.5 |
| Sun LQ et al, 2020 | 19.9 (15.3-25.0) | 98.9 |
| Zhang L et al, 2021 | 19.4 (14.9-24.4) | 98.9 |
| Cheng J et al, 2021 | 19.9 (15.2-25.0) | 98.9 |
| Tu W et al, 2022 | 20.2 (15.6-25.3) | 98.9 |

**Supplementary Figure 1. Forest plot of subgroup analyses**

**(A)**


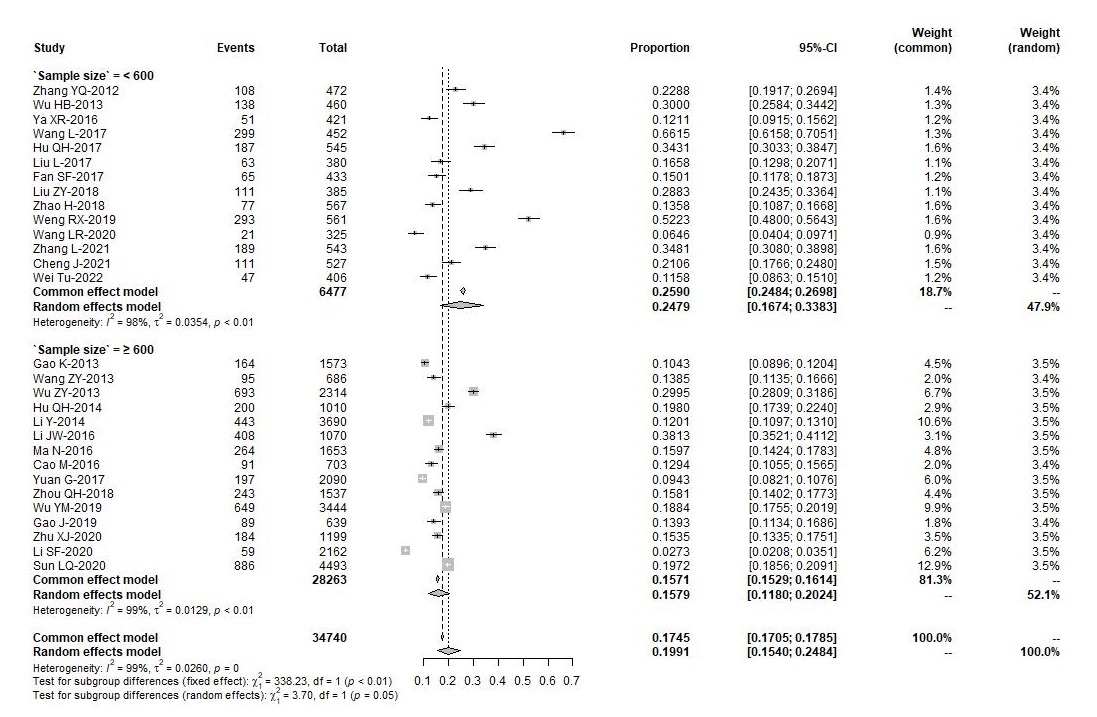


Forest plots of pooled prevalence of syphilis among people living with HIV by sample size

**(B)**


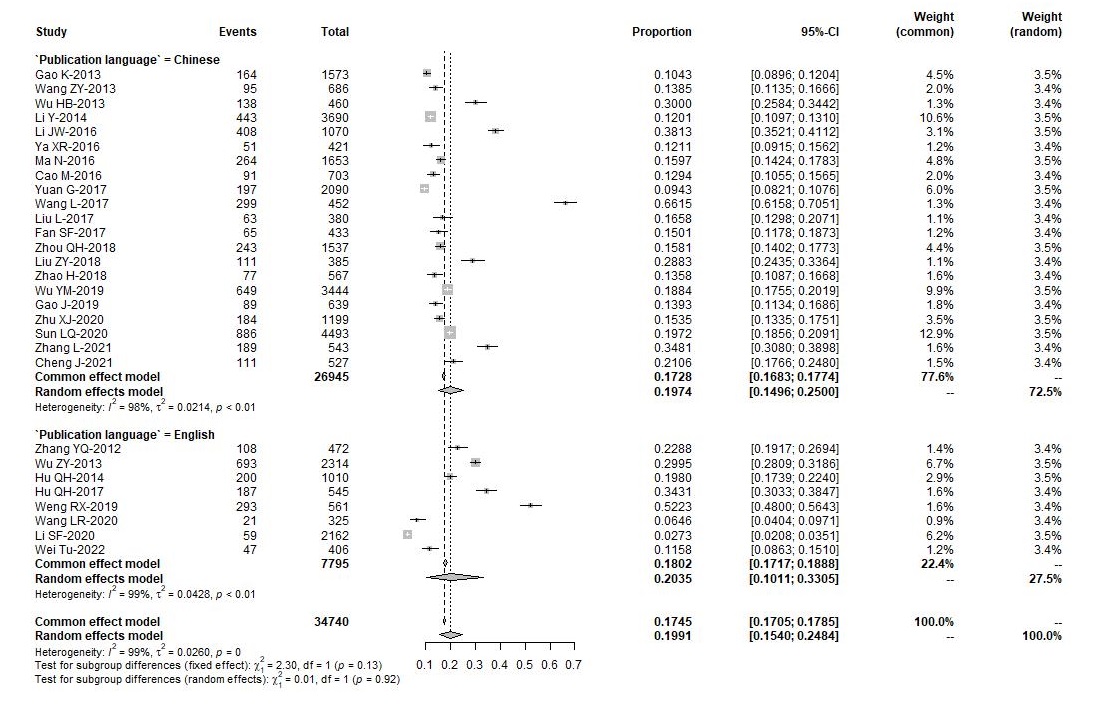


Forest plots of pooled prevalence of syphilis among people living with HIV by publication language

**(C)**


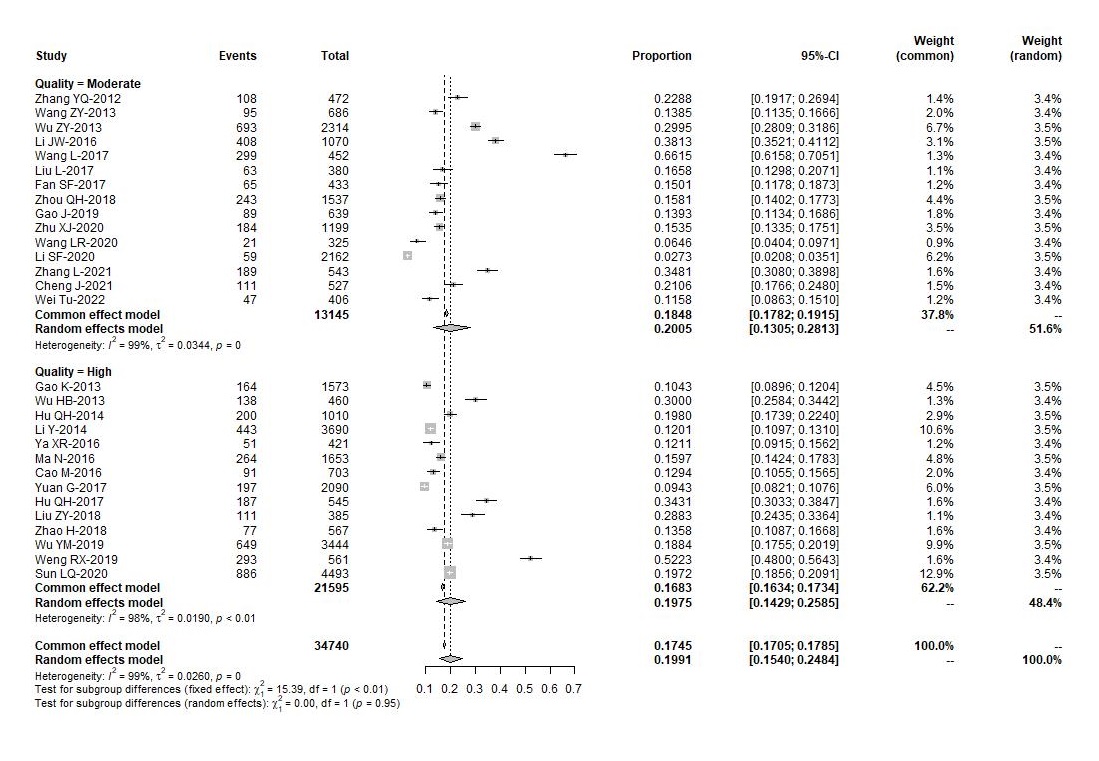
Forest plots of pooled prevalence of syphilis among people living with HIV by study quality

**(D)**


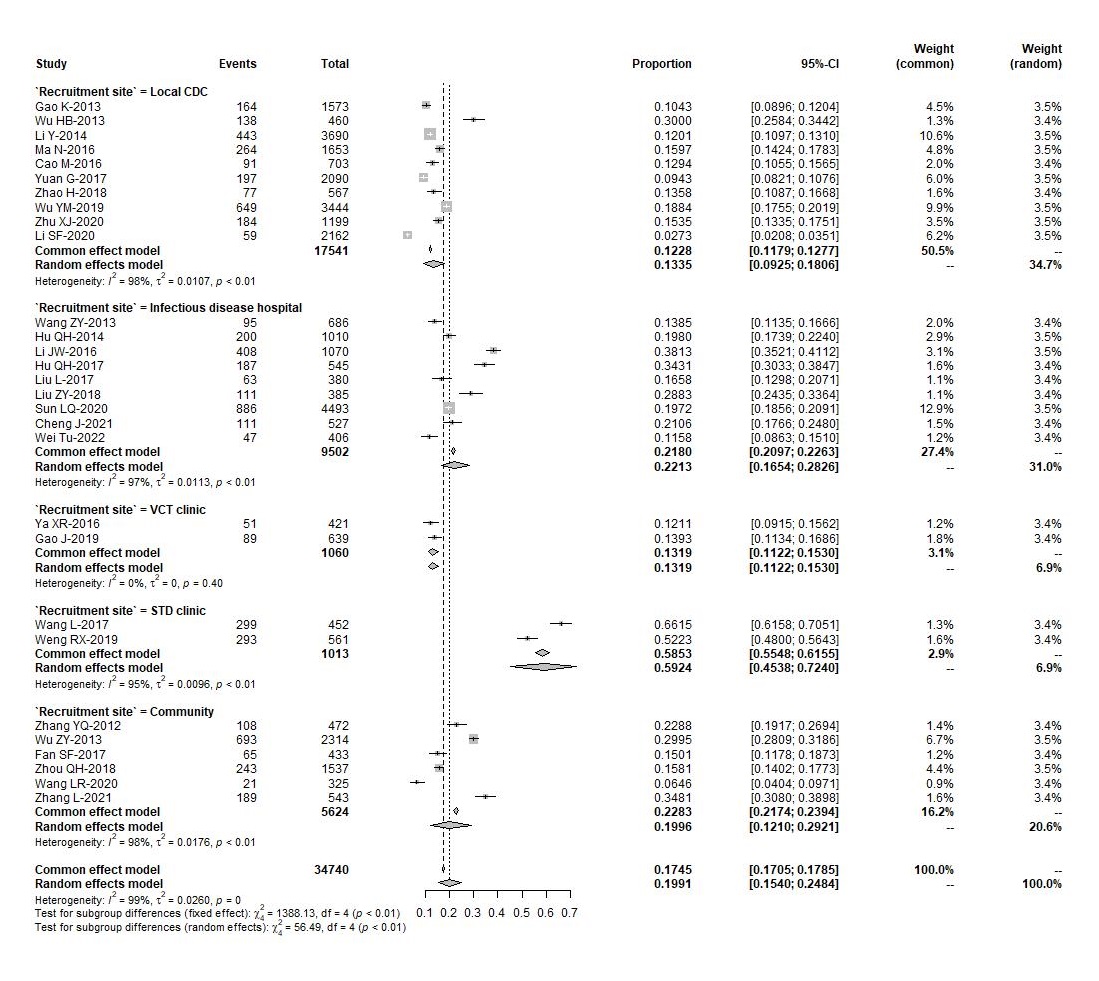


Forest plots of pooled prevalence of syphilis among people living with HIV by recruitment site

Abbreviations: CDC, Center for Disease Control and Prevention; STD, sexually transmitted disease; VCT, voluntary counseling and testing.

**(E)**


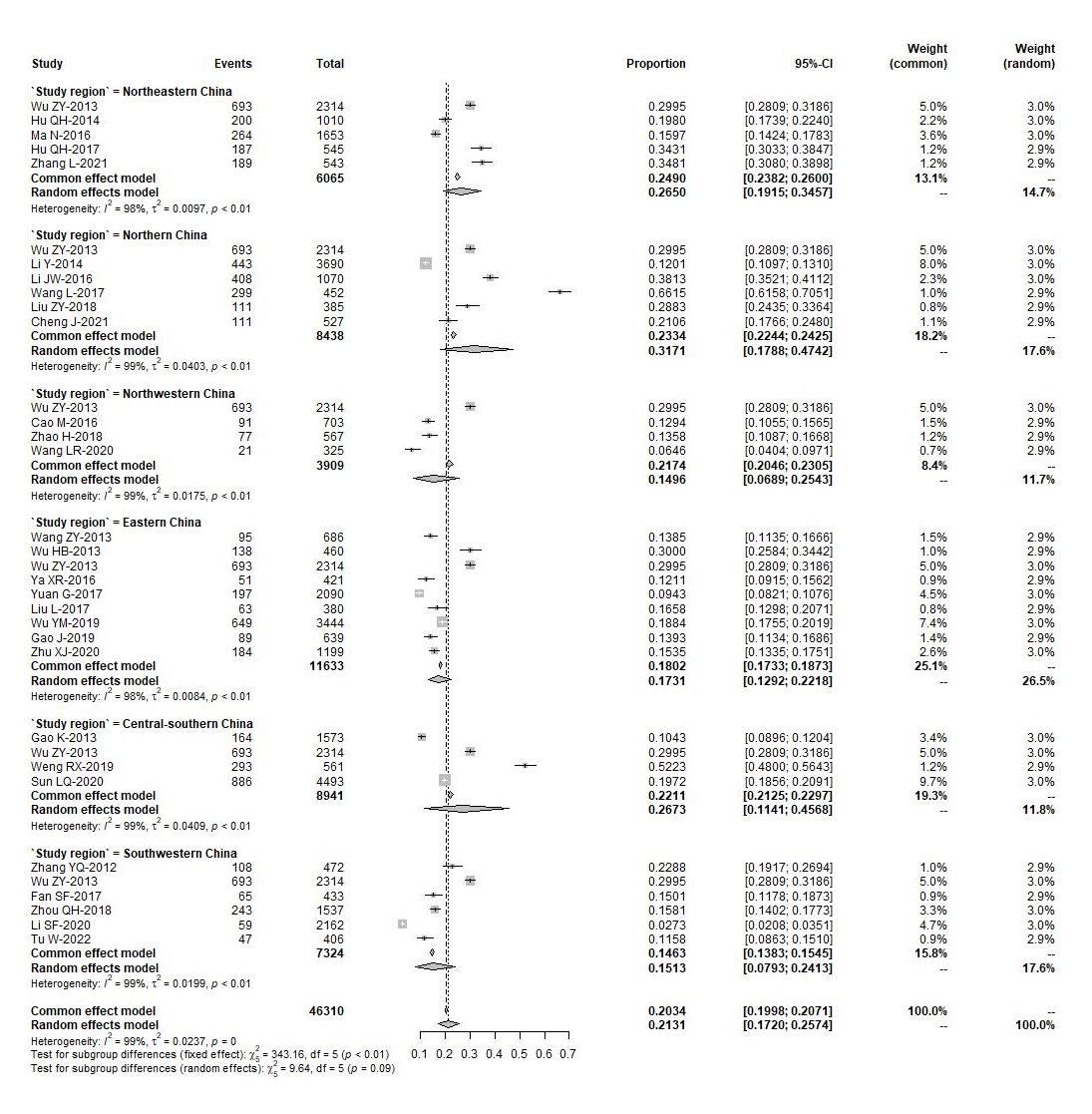


Forest plots of pooled prevalence of syphilis among people living with HIV by study region

**(F)**


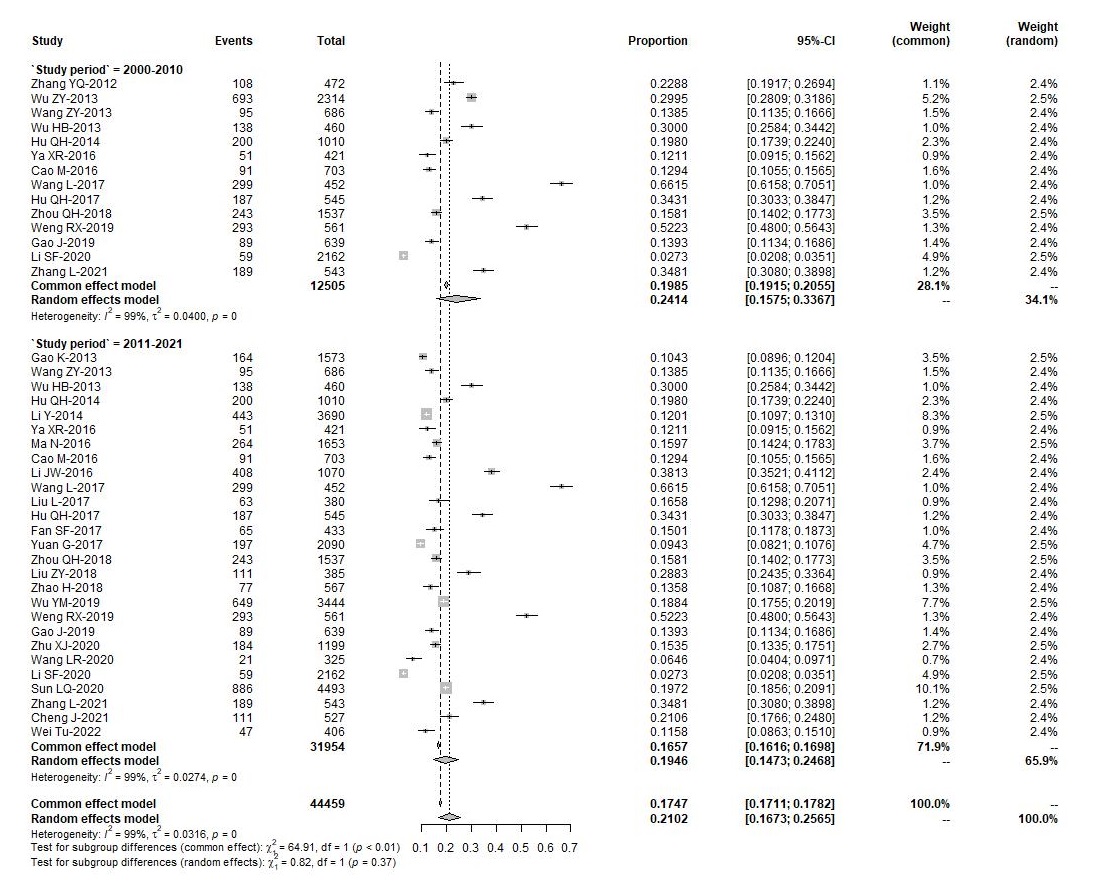


Forest plots of pooled prevalence of syphilis among people living with HIV by study period

**(G)**


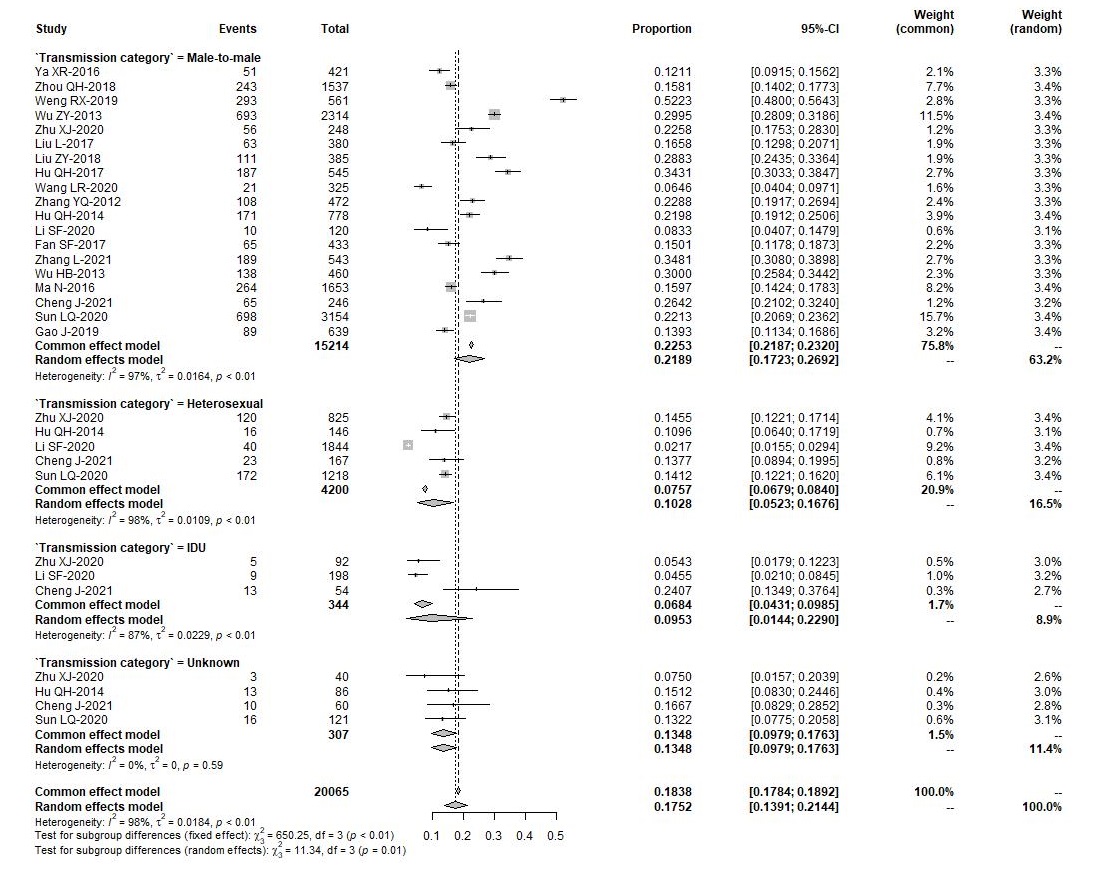


Forest plots of pooled prevalence of syphilis among people living with HIV by transmission category

IDU indicates intravenous drug use.

**(H)**


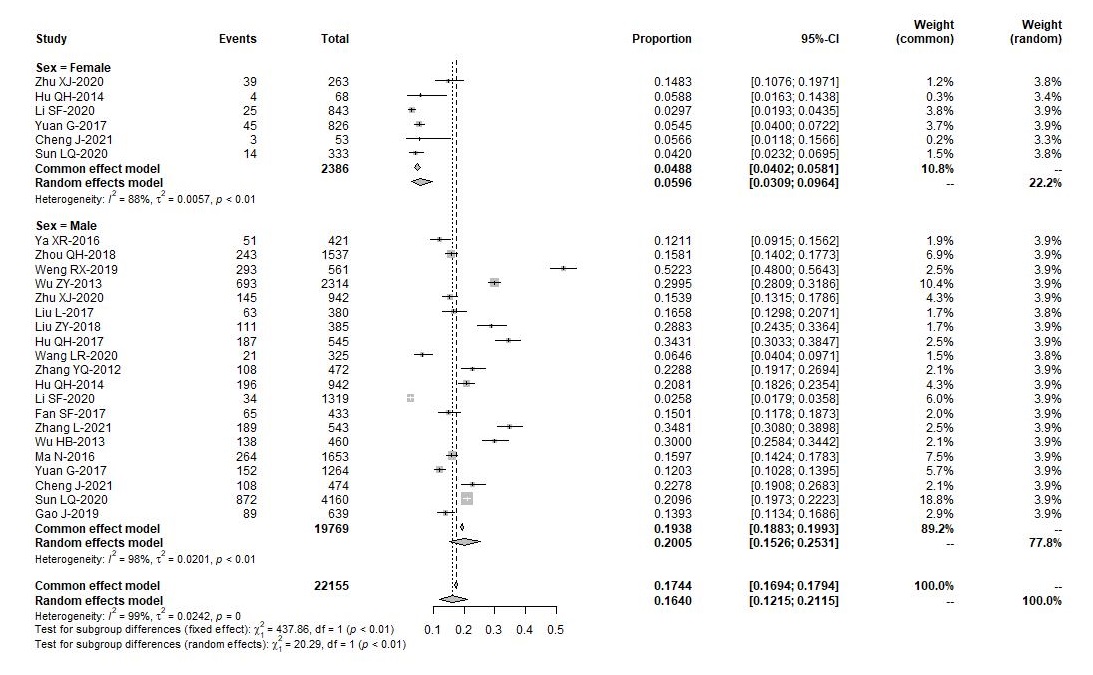


Forest plots of pooled prevalence of syphilis among people living with HIV by sex

**(I)**


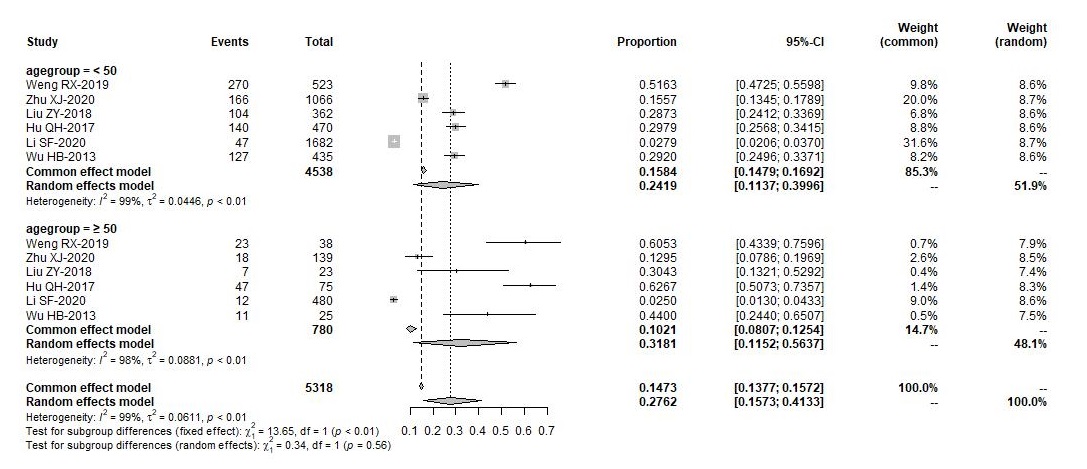


Forest plots of pooled prevalence of syphilis among people living with HIV by age

**(J)**


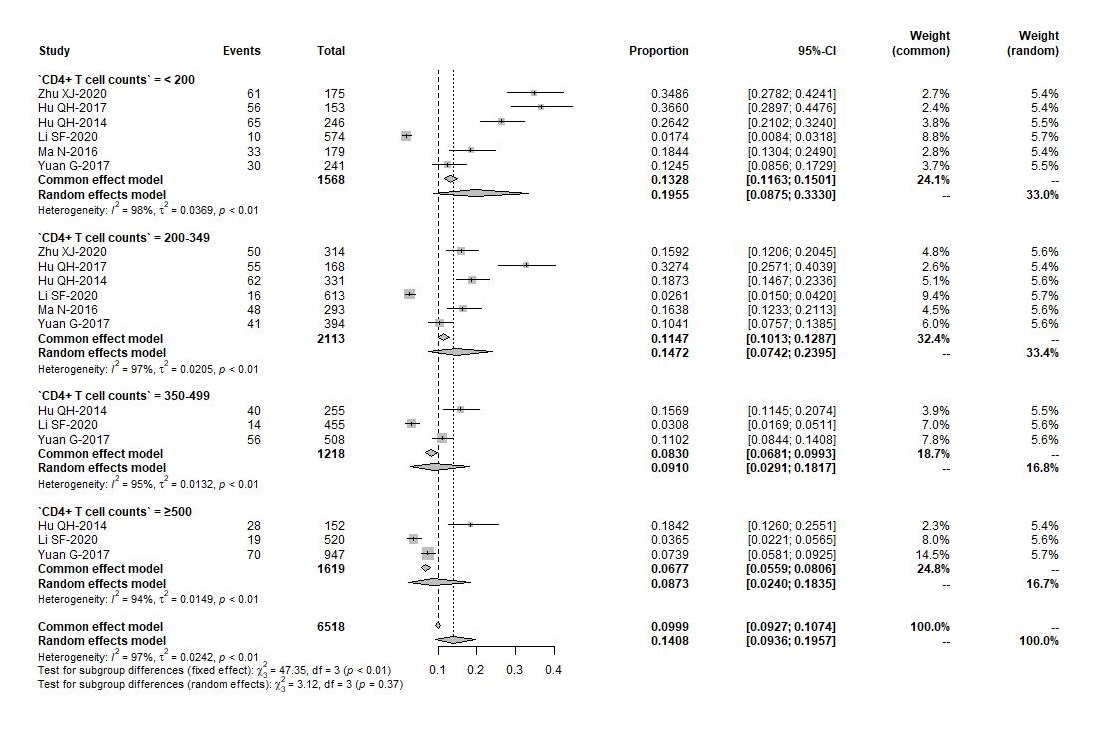


Forest plots of pooled prevalence of syphilis among people living with HIV by CD4+ T cell count

**(K)**


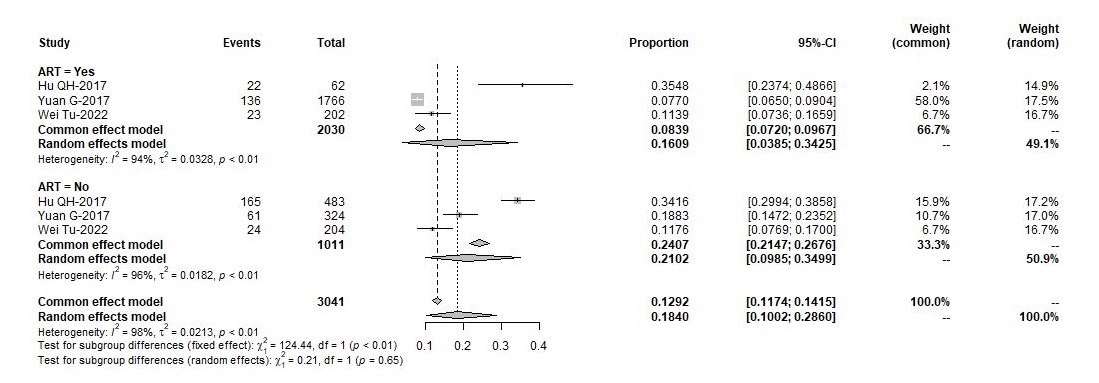


Forest plots of pooled prevalence of syphilis among people living with HIV by ART

ART indicates antiretroviral therapy.

REFERENCE

1. Loney PL, Chambers LW, Bennett KJ, Roberts JG, Stratford PW. Critical appraisal of the health research literature: prevalence or incidence of a health problem. *Chronic Dis Can*. 1998 (19):170-6.
